# Polyphenols: Chemoprevention and therapeutic potentials in hematological malignancies

**DOI:** 10.3389/fnut.2022.1008893

**Published:** 2022-10-26

**Authors:** Ogochukwu O. Izuegbuna

**Affiliations:** Department of Haematology, Ladoke Akintola University of Technology (LAUTECH) Teaching Hospital, Ogbomoso, Nigeria

**Keywords:** polyphenols, hematological malignancies, signaling pathways, apoptosis, immunomodulation, combination therapy, clinical trials

## Abstract

Polyphenols are one of the largest plant-derived natural product and they play an important role in plants’ defense as well as in human health and disease. A number of them are pleiotropic molecules and have been shown to regulate signaling pathways, immune response and cell growth and proliferation which all play a role in cancer development. Hematological malignancies on the other hand, are cancers of the blood. While current therapies are efficacious, they are usually expensive and with unwanted side effects. Thus, the search for newer less toxic agents. Polyphenols have been reported to possess antineoplastic properties which include cell cycle arrest, and apoptosis via multiple mechanisms. They also have immunomodulatory activities where they enhance T cell activation and suppress regulatory T cells. They carry out these actions through such pathways as PI3K/Akt/mTOR and the kynurenine. They can also reverse cancer resistance to chemotherapy agents. In this review, i look at some of the molecular mechanism of action of polyphenols and their potential roles as therapeutic agents in hematological malignancies. Here i discuss their anti-proliferative and anti-neoplastic activities especially their abilities modulate signaling pathways as well as immune response in hematological malignancies. I also looked at clinical studies done mainly in the last 10–15 years on various polyphenol combination and how they enhance synergism. I recommend that further preclinical and clinical studies be carried out to ensure safety and efficacy before polyphenol therapies be officially moved to the clinics.

## Introduction

Hematological malignancies can be defined as a heterogenous group of cancers of blood cells and blood-forming tissues such as bone marrow and lymph nodes. They can be classified as leukemia (acute and chronic), lymphoma (Non-Hodgkin and Hodgkin) and myeloma. According to the GLOBOCAN 2020 report, hematological malignancies accounted for more than one million cancer cases (1). The diversity of their incidence and pathogenesis depends on their subtypes, which are broadly classified as lymphoid and myeloid according to the world health organization (WHO) classification of tumors of hematopoietic and lymphoid tissue (2). More than 400,000 cases and 300,000 deaths of leukemia were reported in 2018 and 2020 (1, 3).

The incidence of hematologic malignancies (HM) varies across regions and is also based on subtypes, age, gender, co-morbidities and socioeconomic status. While the incidence rate of some HM like chronic myeloid leukemia (CML) has decreased, the incidence of others like chronic lymphocytic leukemia (CLL) has increased in some countries (4). Co-morbidities like HIV increase the risk of HM (5). In the modern era of effective anti-retroviral therapy, the incidence rate of HM is still higher, and the 5-year survival rate is also significantly lower than the general population (6).

For the past few decades, most especially in the 21st century, there has been an explosion of knowledge and innovative technologies in the field of oncology which has resulted in newer and more effective therapies, especially in the field of hemato-oncology. Some of these recent therapies are targeted therapies, which make use of synthetic molecules and antibodies to target specific protein molecules and receptors in tumor growth and signaling pathways. This in most cases leads to fewer off-target activities and adverse effects. There are, however, the cost-to-benefit issues. Barnes and colleagues reported that ibrutinib a novel oral Bruton’s tyrosine kinase (BTK) inhibitor that has shown significant efficacy in the management of CLL was not cost-effective as initial therapy (7). The targeted therapies such as BTK inhibitors have shown clinical benefits in some groups of patients e.g., patients with del17p, and are used most often in these groups of patients, but response rates vary. Idelalisib an oral phosphatidylinositol 3-kinase delta isoform (PI3Kδ) has shown substantial activity in patients with CLL, however, the complete remission rate is comparatively low. In a clinical trial study of treatment-naïve older patients (median age, 71 years) with CLL treated with idelalisib and rituximab, the overall response rate (ORR) was 97% and the complete response rate was 19% (8). Acalabrutinib is a selective, next-generation covalent BTK inhibitor in another trial was shown to have an overall response rate was 97% and a complete response of 7% (9). The complete remission rate of the targeted therapies mentioned in CLL is low compared to a chemoimmunotherapy regimen with a complete remission rate of 72% (10). While most of the targeted therapies are target-specific e.g., Bcr-Abl oncoprotein in CML, most tumors are known to activate multiple signaling pathways and adopt/facilitate various resistance mechanisms to targeted drugs. Chemotherapies on the other hand, due to their adverse toxicities which often are severe and reduce the quality of life of patients are avoided in certain clinical settings. Such adverse effects include hematological toxicity, nephrotoxicity, hepatotoxicity, neurological toxicity, etc. Indeed chemotherapies are being systematically phased out.

Due to the challenges posed by these treatments natural products such as polyphenols are being regarded as ideal alternatives with comparable efficacy, safety and less toxicity profiles (11, 12). In a concerted effort at finding directed at finding alternative treatment options in HM, phytochemicals most especially polyphenols provide some interesting applications in this regard. Phytochemicals are plant-derived compounds that have been used in the prevention and treatment of many diseases. They are non-nutrient bioactive chemical compounds produced by plants to enhance their resistance to microbes as well as aid the repulsion of some predators (13). Polyphenols have been extensively studied both *in vivo* and *in vitro* in different cancers (14, 15). They are a large family of about 10,000 compounds having at least one aromatic ring with one or more hydroxyl functional groups attached (14). Natural polyphenols are a large group of plant secondary metabolites ranging from small molecules to highly polymerized compounds (16). They are biologically active compounds with activities against various chronic diseases. They are readily found in foods and beverages of plant origin including fruits, vegetables, spices, soy, nuts, coffee, tea, and wine). Regular consumption of polyphenol-rich diets has been associated with many health benefits. This includes a reduction in cardiovascular events (17, 18), modulation of anti-inflammatory pathways (19), and also in cancer prevention (20).

While their activities are in no doubt, the major challenge to their use in clinical practice is their low oral bioavailability. This is a result of low stability and poor pharmacokinetics which limits their bioavailability when they undergo hepatic phase I/II metabolism before reaching systemic circulation. Thus, the development of a delivery system that favors improved biological activities of polyphenols with better stability is of utmost importance for their clinical use. The activities of polyphenols in HM reveal some interesting applications especially their ability to modulate several signaling pathways such as PI3K and key proteins like NF-kB. The objective of this review is to discuss the chemistry, and biological activities of polyphenols on HM and explore some delivery systems that can enhance their efficacy for use in clinical practice.

## Chemistry of polyphenols

**FIGURE 1:**
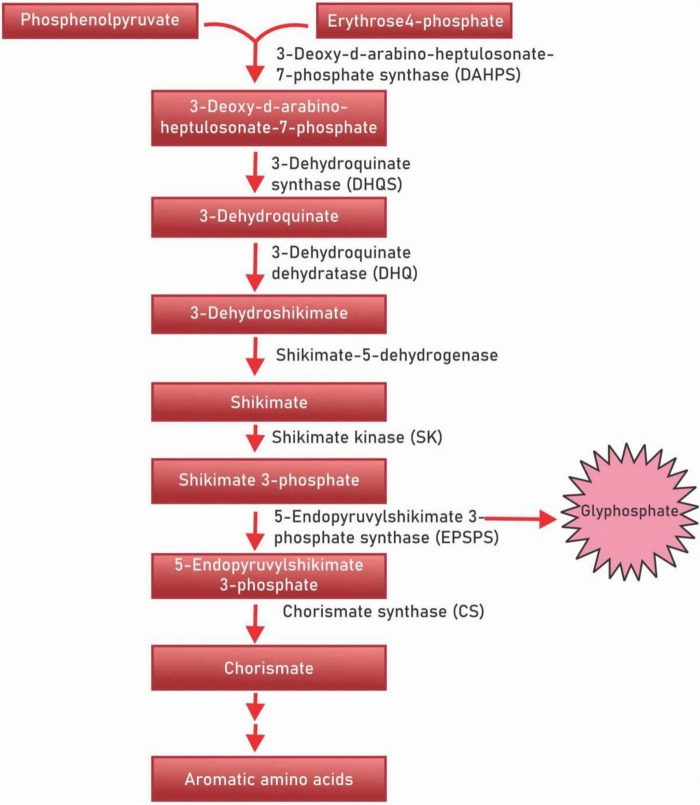
Shikimic acid pathway.

Polyphenols are a diverse class of secondary metabolites that are derivatives of shikimic acid and phenylpropanoid – the shikimate biosynthesis pathway (Figure 1). This biochemical pathway serves for the production of polyphenolic compounds in bacteria, fungi and plants by converting the simple carbohydrate molecules (resulting from the pentose phosphate pathway and glycolysis) into phenylalanine and tryptophan (21). Shikimic acid is named after the highly toxic Japanese *shikimi* (*Illicium anisatum*) flower from which it was first isolated (22). Shikimic acid is a key intermediate of the shikimate biosynthesis pathway and acts as a precursor in the synthesis of the drug oseltamivir phosphate (Tamiflu), a neuraminidase inhibitor that acts against such viruses as the avian influenza virus H5N1, and the human influenza virus H1N1 (23). The shikimate pathway in micro-organisms is responsible for the production of aromatic amino acids L-phenylalanine (L-Phe), L-tyrosine (L-Tyr), and L-tryptophan (L-Trp) (24, 25). However, in plants, these aromatic acids though important for protein synthesis, also serve as precursors for diverse secondary metabolites that are important for plant growth (26). The principal aromatic phenolic compounds synthesized from L-Phe and L-Tyr are cinnamic acids and esters, coumarins, phenylpropenes, chromones (C_6_-C_3_), stilbenes, anthraquinones (C_6_-C_2_-C_6_), chalcones, flavonoids, isoflavonoids, neoflavonoids (C_6_-C_3_-C_6_), and their dimers and trimers, respectively (C_6_-C_3_-C_6_)_2_,_3_, lignans, neolignans (C_6_-C_3_)_2_, lignans (C_6_-C_3_)_*n*_, aromatic polyketides, or diphenylheptanoids (C_6_-C_7_-C_6_).

## Flavonoids

**FIGURE 2:**
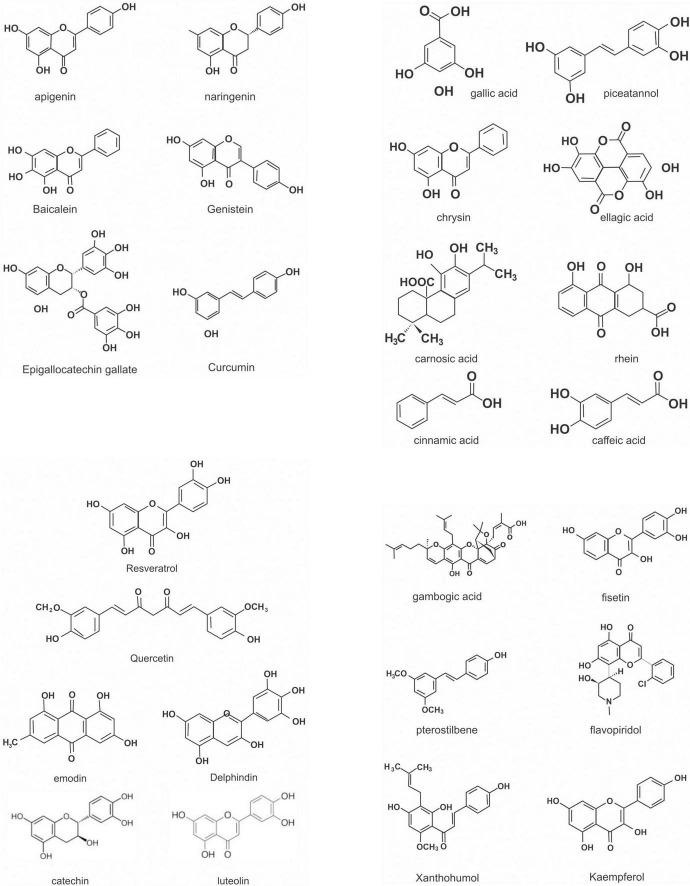
Chemical structures of some polyphenols.

Flavonoids are a large class of polyphenolic secondary metabolites found in fruits, grains, vegetables, flowers, and certain beverages. They play a variety of roles in plants, and are responsible for the color and aroma of flowers and fruits as well as protect plants from different biotic and abiotic stresses especially ultraviolet (UV) light (27). They may also function against frost hardiness, drought resistance, heat acclimatization and freezing tolerance (28, 29). Thus, they have potential applications in the nutraceutical, pharmaceutical, cosmetic and biotechnology industries. Flavonoids can be divided into six subclasses based on chemical structures: anthocyanidins, flavanones, flavonols, isoflavones, flavone, and flavan-3-ol (30) (Figure 2 and Table 1). The glycosylated flavonols are the most widely distributed in the diet (31). On the other hand, flavonoids account for about 60% of all natural polyphenols (14).

**TABLE 1:** The major classes of dietary flavonoids.

Class	Types	Sources
Flavones	Apigenin, baicalein	Celery, thyme, parsley, chamomile
Flavonols	Quercetin, kaempferol	Onions, kale, cucumbers, raspberries
Flavan-3-ols	Epigallocatechin gallate, catechin	Green tea, berries, apricot, red wine
Flavanones	Naringenin, Hesperetin	Grape fruits, oranges, lemon
Isoflavones	Genistein, daidzein	Soybeans, raisins, nuts, lentils
Anthocyanidin	Delphindin, cyanidin	Black berries, pomegranates

Currently, there are about 15,000 naturally occurring flavonoid compounds (31). Flavonoids generally consist of a benzopyrone core skeleton which is characterized by the presence of 15 carbon atoms as the base skeleton, organized in the form C6–C3–C6 (A + C – B) (two benzenic rings A and B) and linked by a unit of three carbons that may or not form a third-ring structure (pyran ring C). Flavonoids occur in various forms in nature; they come as either O-glycosides or C-glycosides which play a role in their bioactivities (32). They also occur as aglycones or can be hydroxylated or methylated (33).

### Flavones

Flavones are a class of flavonoids commonly found in some food and fruits giving a yellow or orange color. Their chemical structure is characterized by a double bond between C3 and C4, a keto group at C4, and no substitution in C3. Flavones have emerged as important metabolites that are involved in plant signaling and defense (34). They are also involved in protection against UV light (35) and oxidative stress (36); allelopathy (37); lignification (38) and pathogen resistance (39). Some of the better-known flavones include luteolin, wogonin, apigenin, tangeretin and chrysin.

4’,5,7-Trihydroxyflavone, also known as apigenin which can be synthesized through a two-step pathway is present in black and green tea (40). Apigenin has been shown to have some good activities against leukemia cell lines including suppression of cell proliferation, induction of cell cycle arrest and induction of apoptosis in leukemia cell lines (41, 42). Also, apigenin when combined with etoposide or cyclophosphamide-induced apoptosis via the mitochondrial pathway, increases the expression of pro-apoptotic cytochrome *c*, SMAC/DIABLO, and HTRA2/OMI, which promoted caspase-9 and -3 activation (43). Interestingly, apigenin has low intrinsic toxicity to normal cells.

### Flavonols

Flavonols are a class of flavonoids that have the 3-hydroxyflavone backbone; having a double bond between positions 2 and 3 and an oxygen (a ketone group) in position 4 of the C ring, like flavones from which, however, they differ in the presence of a hydroxyl group at the position 3 (IUPAC name: 3-hydroxy-2-phenylchromen-4-one). They are distinct from flavanols like catechins. They are colorless molecules found mainly in the skin and leaves of fruits and vegetables since their biosynthesis is stimulated by light. The majority of flavonols exist as O-glycosides and rarely as C-glycosides (44). They are also very diverse in methylation and hydroxylation patterns along with flavones; they are perhaps the largest subgroup of flavonoids in fruits and vegetables (27). Some fruits and vegetables rich in flavonol include elderberry juice, rocket lettuce, red onions, fresh cranberries, fresh figs, apples, fresh capers, dried parsley and tea. The consumption of flavonols is found to be associated with a wide range of health benefits including antioxidation (45), anti-inflammatory (46), and anti-obesity (47) and reduced risk of vascular disease. The major flavonols that are well-studied include kaempferol, quercetin, fisetin, isorhamnetin, and myricetin. Recent studies have shown that flavonol has good anticancer activities including against leukemia (48). Quercetin has been shown to induce cell death via downregulation of VEGF/Akt signaling pathways and mitochondria-mediated apoptosis in AML cells (49). The cell death is caspase-dependent apoptosis, and this also depends on the decrease of mitochondria membrane potential (MMP) and Bcl-2 proteins induced by quercetin. Kaempferol was shown to decrease cell viability in tested acute promyelocytic cell lines with an associated decrease in Akt, BCL2, ABCB1, and ABCC1 genes expression, while the expression of CASP3 and BAX/BCL-2 ratio were significantly increased (50). Recently, an O-methylated flavonol was shown to target multiple kinases that play critical roles in survival signaling in AML, including FLT3, MNK2, RSK, DYRK2 and JAK2 (51). Thus, it can be developed as a novel therapeutic for drug-resistant acute myeloid leukemias.

### Flavan-3-ol

Flavan-3-ol also known as flavanol or dihydroflavonols are the 3-hydroxy derivatives of flavanones. Flavan-3-ol are considered the most complex subclass of flavonoids, ranging from the simple monomers to the oligomeric and polymeric proanthocyanidins. In the monomeric form, they have two chiral centers at C2 and C3 which give rise to four isomers for each level of B-ring hydroxylation (52) and also the absence of a double bond between C-2 and C-3. Unlike other flavonoids, they rarely exist as glycosides in plants (53) flavanols are found in common foods, including cereals, legumes, fruits, vegetables, forages, hops, beers, red wine, tea, cocoa, grapes, and apples. They are known to exhibit health benefits including acting as antioxidants, anticancer, cardioprotective, anti-microbial, anti-viral, and neuroprotective agents. Some of the well-known flavan-3-ol include: (+)-catechin; (+)-gallocatechin; (–)-epicatechin; (–)-epigallocatechin; (–)-epicatechin 3-gallate; (–)-epigallocatechin 3-gallate; theaflavin; theaflavin 3-gallate; theaflavin 3′-gallate; theaflavin 3,3′-digallate; and thearubigins (54). A few of the health benefits of flavan-3-ol include: acute promyelocytic cell lines treated with various concentrations of catechin significantly reduced their proliferation, and induced cell apoptosis, in association with mitochondria damage, ROS production and caspase activation (55). Epigallocatechin 3-gallate (EGCG) inhibited multiple myeloma cell line U266 proliferation and induced apoptosis by targeting EZH2 and modulating the mitochondrial apoptosis pathway (56). In a similar experiment, EGCG treatment reversed leucocytosis, anemia and thrombocytopenia, and prolonged survival of PML/RARα mice; in combination with all-trans retinoic acid (ATRA) yielded increased expression of CD15 marker (57).

### Flavanones

Flavanones are an important group of flavonoids also called dihydroflavones. They have two benzene rings, A–B bound by a dihydropyrone ring C, with chirality at C3 of the C ring, and no double bond between C-2 and C-3 which is the only difference between flavones and flavanones (27). They occur mainly as the *S*- or (–)-enantiomer with the C-ring attached to the B-ring at C-2 in the α-configuration (58). Like some other groups of flavonoids, flavanones also do occur as hydroxyl, glycosylated, and *O*-methylated derivatives. They are generally found in almost all citrus fruits and are responsible for the bitter taste of their juice and peel (59). Some examples of flavanones include Hesperitin, naringenin and eriodictyol. Flavanones found in citrus have some pharmacological activities including anti-inflammatory (60), and antioxidation (27). Some flavanones have been reported to possess anticancer properties through the regulation of some key pathways (61).

### Isoflavones

Isoflavones are a distinct group of flavonoids that have the B-ring attached at C-3 rather than at the C-2 position of the pyran ring, a feature that distinguishes them from flavones are found almost exclusively in leguminous plants where they play a role in plant-microbe interactions (62). Isoflavones are also known to act as phytoalexins in plants i.e., compounds produced by the plants during stress or pathogen attacks (63). They are often referred to as phytoestrogens because of their similarity to 17-β-estradiol. Isoflavones may occur as aglycons or as glycosides (64), but their biological activity is from their aglycones (65). Sources of isoflavone include soybeans, chickpeas, fava beans, pistachios, peanuts, and other fruits and nuts (66). Examples of isoflavone include Genistein (7,4’-dihydroxy-6-methoxy isoflavone), daidzein (7,4’-dihydroxyisoflavone), glycitein (7,4’-dihydroxy-6-methoxy isoflavone), biochanin A (5,7-dihydroxy-4’-methoxy isoflavone), and formononetin (7-hydroxy-4′-methoxy isoflavone). Isoflavones are known to have health benefits which can also be seen in the increased number of isoflavone-containing nutritional health products. Some of these health benefits include the prevention of osteoporosis (59, 67), cardiovascular diseases (68), antioxidation and anti-inflammatory (69). It also has chemopreventive and chemotherapeutic roles, especially in hormone-dependent cancers (70). In a recent meta-analysis, the consumption of soy isoflavones was reported to reduce the risk of breast cancer in pre-menopausal and post-menopausal women (71). Genistein and daidzein inhibited cell migration, invasion, proliferation and sphere formation, and induced cell cycle arrest and apoptosis in metastatic ovarian cancer models (72). Genistein is also reported to have an antiproliferative effect on leukemia (73), lymphoma (74) and myel2oma (69, 75).

### Phenolic acids

Phenolic acids are aromatic acids consisting of an aromatic ring with one or more hydroxy or methoxy groups. Phenolic acids are divided into two major subgroups: hydroxybenzoic and hydroxycinnamic acid. Hydroxycinnamic acid is more abundant than hydroxybenzoic acid. Hydroxycinnamic acids are secondary metabolites derived from phenylalanine and tyrosine and they all have a C6C3 carbon skeleton with a double bond in the side chain that may have a cis or a trans configuration. They may be present as free carboxylic acids or in bound forms as amides, esters or glycosides (76). Hydroxycinnamic acids share a similar pathway of production with the likes of lignins, 89 coumarins, lignans, stilbenes, chalcones, anthocyanins and flavonoids (77). They are well-distributed in most plants including many species that are consumed as food or processed into beverages. They are abundant in fruits, vegetables, cereals, legumes, soybeans, coffee, and tea (77, 78). The most common hydroxycinnamic acids are ferulic, caffeic, *p*-coumaric, and sinapic acids (79). On the other hand, hydroxybenzoic acids have a general structure of C_6_-C_1_. They can be found in some foods like red fruits, onions and black radish, etc. (21). Examples of hydroxybenzoic acids are gallic, vanillic, syringic, 2,3-dihydroxybenzoic acid (Pyrocatechuic acid), 2,5-dihydroxybenzoic acid (Gentisic acid), 3,4-dihydroxybenzoic acid (Protocatechuic acid), 3,5-dihydroxybenzoic acid (α-Resorcylic acid) and 3-monohydroxybenzoic acid (33, 80). Phenolic acids have many health benefits. These include anti-inflammatory and antioxidative actions (81, 82), antidiabetic (83), and hepatoprotective (84), and antineoplastic (85, 86). Caffeic acid (3,4-dihydroxycinnamic acid) phenethyl ester (CAPE) is reported cytotoxic and anti-proliferative actions on RPMI 8226, H929, U266 and ARH77 cell lines, and also synergises with bortezomib in growth inhibition and reduction of NF-kB binding activity and IL6 levels (87). In preclinical studies, caffeic acids and its analogues have also been reported to downregulate specificity protein 1 and IKZF1-IRF4-MYC axis in myeloma cells including cell lines resistant to immunomodulatory drugs lenalidomide and pomalidomide (88). Gallic acid (3,4,5-trihydroxy benzoic acid) was observed to significantly induce apoptosis in AML cell lines via a caspase-dependent pathway in a dose-dependent manner and augment some chemotherapy agents’ efficacy (89). It is also reported to induce apoptosis in the Jurkat cell line (90).

### Stilbenoids

Stilbenoids are non-flavonoid polyphenols just like the lignans, coumarins and xanthones (91). They are hydroxylated derivatives of stilbene with a C_6_–C_2_–C_6_ structure. Stilbenoids can be either monomers or polymers. They do exist as aglycones or glycosidic conjugates and can be further processed by methylation, glucosylation and prenylation (92). Like isoflavones, stilbenoids are regarded as plant phytoalexins (92, 93). They are found in some foods such as grapes, rhubarb, passion fruit, berries, white tea and red wine (94, 95). Stilbenoids demonstrate various health benefits, including antioxidant, anti-inflammatory (96), anti-microbial activities (97) and anti-neoplastic (98, 99). In leukemia, a stilbenoid tyrosine kinase inhibitor was once reported to inhibit the proliferation of the Jak2-V617F expressing human erythroleukemia in a caspase-dependent manner as well as the cleavage of PARP (100).

## Molecular activities of polyphenols

From a clinical point of view, hematological malignancies (HM) are generally incurable. While therapeutic options have improved, disease relapse and resistance are rather not uncommon. Chemotherapy and immunotherapy remains the mainstay of treatment, but not without their associated side effects. For example, treatment with CD19 chimeric antigen receptor (CAR) T cells the most recent approved innovative therapy for patients with lymphoid malignancies, especially with relapsed/refractory disease (101) is not without serious adverse effects. Its high therapeutic response rate is accompanied by serious side effects such as cytokine release syndrome (CRS) and severe neurotoxicity termed immune effector cell-associated neurotoxicity syndrome (ICANS) (102). In a recent multicenter observational study of patients treated with CD19-targeted CAR T-cell therapy for relapsing lymphoma, 43% developed neurotoxicity and more than half of the patients (64%) had grade 1–2 severity and 34% had grade 3–4; a further 80% developed CRS (103). These side effects along with the fact that some patients do not respond to CAR T cell therapy or relapse after remission underscore the need for newer therapies. One potential source of therapeutics can be polyphenols; albeit some of the pathways and strategies muted to enhance CAR T cell therapy are already known targets of polyphenols (104).

Like many approved antineoplastic drugs, polyphenols target different molecular pathways that are involved in carcinogenesis. Some of these targets are involved in cell signaling, proliferation and survival, cellular stress response, apoptosis, etc. For example, mutations in some components of the NF-kB pathway especially its regulators like NFKB2, TRAF2, TRAF3, CYLD, NFKB1, TACI, NIK, *REL*, *NFKB2*, *IKBA*, *CYLD*, *NEMO*, etc., that are involved in both the canonical and non-canonical pathway plays a role in multiple myeloma development (105, 106). Resveratrol, a stilbenoid is reported to prevent the ubiquitination of NEMO and IKK-mediated NF-κB activation (107), and mangiferin, a xanthone, is observed to cause a decrease in the expression of phosphorylated NF-κB-inducing kinase (NIK) (108). With their various actions similar to other approved drugs, polyphenols represent prospective therapeutic options for hematological malignancies.

## The phosphatidylinositol 3-kinase/protein kinase B pathway

**FIGURE 3:**
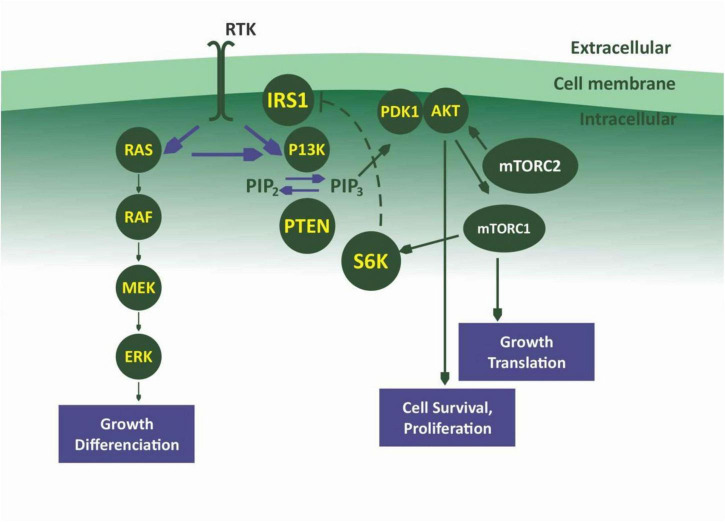
Pl3K/Akt/mTOR pathway.

**FIGURE 4:**
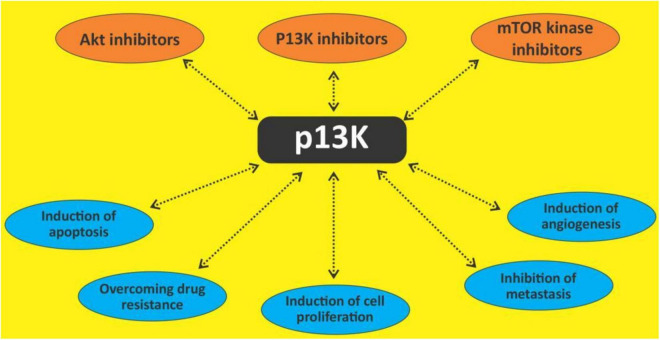
Targeting Pl3K/Akt/mTOR with different outcomes.

The phosphatidylinositol 3-kinase (PI3K)/protein kinase B (Akt) and the mammalian target of rapamycin (mTOR) signaling is one of the most important intracellular pathways. It is involved in the control of many physiological cellular processes as well as the development of malignancies through cell growth, proliferation, and survival (109) (Figures 3, 4). They also play a role in metabolism. The activation of the PI3K/AKT pathway reprograms cellular metabolism through increased activities of nutrient transporters and metabolic enzymes in cancer cells (110). The activation of the PI3K/AKT signaling is downstream of a network of receptor tyrosine kinases (RTKs), cytokine receptors, integrins, and G protein-coupled receptors (GPCRs). Thus, the PI3K is divided into three classes I, II, and III made up of catalytic and regulatory domains. There are four Class I PI3K isoforms subdivided into Class IA PI3K (PI3K α, β, and δ) and class IB PI3K (PI3K γ); three Class II PI3K isoforms (PI3KC2α, C2β, C2γ) and a single Class III PI3K (111). The Class IA are dimers made up of a regulatory subunit p85 (p85α, p55α, p50α, p85β, p55γ), and a catalytic subunit (p110α, p110β, p110δ). The Class IB also a dimer comprise of the regulatory subunits (p101 or p84) and the catalytic subunit p110γ (112, 113). While PI3Kα and PI3Kβ are ubiquitously expressed in different tissues, PI3Kγ is expressed in T lymphocytes (114), whereas PI3K γ is mainly expressed in B lymphocytes and its precursors (115). When PI3K is activated, it stimulates the phosphorylation of its phospholipid substrate phosphatidylinositol 4,5-bisphosphate (PIP_2_) to produce the second messenger phosphatidylinositol 3,4,5-trisphosphate (PIP_3_). PIP_3_ then recruits a subset of signaling proteins with pleckstrin homology (PH) domains to the membrane, including 3-phosphoinositide-dependent protein kinase (PDK1) and AKT, resulting in its phosphorylation at threonine-308 and activation (116).

AKT exist in three isoforms: AKT1, AKT 2, and AKT3. AKT is known to phosphorylate a diverse group of downstream substrates including forkhead box protein O (FOXO), glycogen synthase kinase-3 (GSK-3), and Bcl-2 associated death promoter (BAD). It inhibits the proline-rich AKT substrate of 40 kDa (PRAS40) and tuberous sclerosis complex 2 (TSC2) through inhibition of the GTPase activity of the TSC1/TSC2 complex, thereby activating mTOR complex 1 (mTORC1) through the RAS homologue enriched in brain (RHEB) (117, 118). MTORC exist in two different protein complexes form which are mTORC1 and mTORC2. mTORC1 can be directly inhibited by the natural product rapamycin (119). mTORC1 complex consists of a catalytic subunit mTOR, regulatory-associated protein of mTOR (RAPTOR), mammalian lethal with SEC13 protein 8 (MLST8), and the regulatory proteins PRAS40 and DEP domain-containing mTOR-interacting protein (DEPTOR). mTORC1 plays a key role in cell growth through some substrates that include ribosomal S6 kinase-1 (S6K-1) and eukaryote translation initiation factor 4E binding protein-1 (4EBP-1) (120). mTORC1 also regulate other substrates like unc-51-like autophagy-activating kinase 1 (ULK-1), a key regulator of autophagy, transcription factor EB (TFEB), a regulator of lysosome biogenesis, and Grb-10, an insulin-receptor binding protein (121, 122).

The constitutive activation of the PI3K pathway is rather common in hematological malignancies (123, 124) and certain PI3K isoforms are expressed mainly in hematopoietic cells. This gave room for the development and approval of novel PI3K inhibitors and research into other novel ones.

## Polyphenols and phosphatidylinositol 3-kinase/protein kinase B/mammalian target of rapamycin

The PI3K/Akt/mTOR pathway is seen as a prime target because of its frequent activation in many cancers including hematological malignancies (125, 126). Several *in vitro* and *in vivo* studies have shown that this pathway has direct effects on multiple cellular functions as earlier mentioned. PI3K signaling is reported to affect every step of carcinogenesis, and it is also shown to be a prognostic factor as well as a predictor of response to chemotherapy (127). Thus, this pathway is targeted in various studies using small molecules and natural products (128). Several polyphenols including quercetin, curcumin, resveratrol, apigenin, etc., are known to exert some antineoplastic actions through several mechanisms including the PI3K/Akt/mTOR pathway. The treatment of the flavonoids isorhamnetin, genkwanin and acacetin against some breast cancer cell lines decreased the levels of PI3Kγ-p110, phospho-PI3K, phospho-AKT, phospho-mTOR, phospho-p70S6K, and phospho-ULK in them, thus showing their potential as an inhibitor of the PI3K/Akt/mTOR pathway (129). Currently, there are about four approved PI3K inhibitors (113), two mTORC1 inhibitors (130) and no Akt inhibitor (131) for the management of cancers. While there haven’t been many studies on the effect of polyphenols on the PI3K/Akt/mTOR pathway in hematological malignancies, a few done shows their efficacy. Quercetin has been reported to modulate AKT signaling leading to attenuation of cell survival, inflammation, and angiogenesis in lymphoma-bearing mice (132). Constitutive activation of Akt has been observed in various types of leukemia (133, 134) which is responsible for the anti-apoptotic mechanisms. Apigenin has been noted to inactivate Akt with concomitant down-regulation of Mcl-1 and Bcl-2 which results in apoptosis (42). Curcumin treatment of pre-B ALL cell lines with various translocations induced dephosphorylation of the constitutive phosphorylated AKT/PKB and downregulation of IAPs (135). In primary CLL B cells, curcumin was also observed to inhibit the constitutive activation of pro-survival pathways including Akt (136).

Some of these effects of the attenuation of the PI3K/Akt/mTOR pathway are autophagy and apoptosis.

## Autophagy

Autophagy is a cellular mechanism that leads to intracellular degradation of cell components and organelles through a lysosome-dependent regulated mechanism in order to adapt to metabolic stress and survival (137). Autophagy is controlled by a group of autophagy-related genes (Atg genes) as well as several proteins that play a role in the regulation of initiation of autophagy including mTOR which acts as a sensor for growth factors and nutrient availability. Thus, PI3K/Akt/mTOR pathway is a negative regulator of autophagy (138, 139). Polyphenols are known to induce autophagy in leukemic cells. Resveratrol has been shown to be an autophagic modulator in MOLT-4 and HL-60 cells (140). It also induces autophagy in imatinib-sensitive (IM-S) and resistant (IM-R) K562 cells (141). The polyphenols emodin, cis-stilbene, apigenin and rhein have been reported to induce autophagy of myeloid (K562 cells) and lymphoid leukemia cells (CCRF-CEM) (142). Curcumin has also been shown to have inhibitory effects on leukemia by inducing autophagy. A study by Guo et al. discovered that curcumin induces autophagic cell death in human Philadelphia chromosome-positive acute lymphoblastic leukemia SUP-B15 cells via activating RAF/MEK/ERK pathway (143). Pi3k is known to regulate MEK/ERK signaling (144); ERK and Akt are known to activate MTORC1 signaling thus, promoting autophagy (145). Curcumin use has also been associated with the autophagic death of the CML cell line K562 cells (146). A curcumin derivative has also been shown to induce autophagy in the THP-1 cell line (147). Polyphenols have so far demonstrated the ability to induce autophagy in hematological malignancies.

## Apoptosis

Apoptosis is a form of cell death. It is divided into two, namely: the extrinsic pathway, which is dependent on caspase 8 activation and mediated by death receptors; and the intrinsic pathway which is caspase 9-dependent and mediated by mitochondria (148). Dysregulations have been identified in these two pathways which are associated with pathogenesis, prognosis and resistance to standard chemotherapeutic agents. Several studies have shown that the deregulation of apoptosis is a common and causative event in hematologic malignancies and has prognostic significance (149, 150). The death receptors are members of the tumour necrosis factor receptor (TNFR) family including Fas (CD95), tumor necrosis factor α receptor 1 (TNFR1), tumor necrosis factor α ligand-receptor 1(TRAIL-R1, DR4), tumor necrosis factor α ligand-receptor 2 (TRAIL-R2, DR5), DR3, and DR6. Death-inducing ligands e.g., FasL/CD95 ligand (CD95L), tumor necrosis factor α ligand (TNFα) initiate the extrinsic pathway by interactions with the death receptors. Adaptor proteins are then recruited to the Fas-associated death domain (FADD) and TNF receptor-associated death domain (TRADD) on the death receptor. Inactive forms of some caspase protease families (procaspase 8 and 10) are recruited, forming a “death-inducing signaling complex” (DISC), and resulting in the activation of caspases 8 and 10 (151). There is also the activation of caspase 3, 6, and 7 which lead to apoptotic cell death (148).

The intrinsic pathway on the other hand is a form of regulated cell death initiated by a balance between the proapoptotic and anti-apoptotic BCL-2 family proteins within mitochondria. A series of molecular events involving intrinsic stimuli and BCL-2 family proteins form the mitochondrial outer membrane permeabilization (MOMP) complex resulting in the release of cytochrome c, a second mitochondria-derived activator of caspase (SMAC) and mitochondrial serine protease (Omi). The release of cytochrome c leads to its binding of apoptotic protease-activating factor-1 (APAF-1) and dATP, to form an apoptosome which in turn activates caspase 9. In the process of apoptosome formation, SMAC and Omi inhibit inhibitors of apoptosis proteins (IAP) which are endogenous inhibitors of caspase function (152, 153). Activation of apoptotic caspase 9 shall then lead to the activation of downstream “executioner” caspases.

The complex nature of apoptosis requires that it be closely regulated. Several signaling pathways have been shown to impact apoptosis. The most notable is the phosphatidylinositol 3′-kinase (PI3K) pathway (154). Activated PI3K activates PKB/Akt which leads to the expression of anti-apoptotic genes through the activation of nuclear factor κB (NF-κB) (155). It also influences pro-apoptotic gene expression by inactivating the forkhead superfamily transcription factors AFK and FKHRL1. Activation of Akt is known to inhibit apoptosis through the upregulation of bcl-2 expression (156, 157) and the inhibition of bad (158, 159). Another regulator of apoptosis is the extracellular signal-regulated kinase 1/2 (ERK1/2) signaling pathway which regulates the activity of the bcl-2 family of proteins (160). It is also involved in the ubiquitination of pro-apoptotic proteins BIM, BAD, BIK, etc., for degradation (161, 162).

Several polyphenols have been shown to induce apoptosis in hematological cancers (163). Curcumin treatment of B Pre-ALL cell lines causes downregulation of cIAP1 and XIAP (135). Gossypol a polyphenol isolated from the seed, roots, and stem of the cotton plant (Gossypium sp.) and originally used as a herbal drug in China (164) is known as a bcl-2 inhibitor as well as inducing autophagy in Burkitt lymphoma cells (165, 166). Gossypol compounds have hence been tried in some small clinical trials to determine efficacy (167, 168). Piceatannol induces a Fas/FasL upregulation in U937 cells (169). Resveratrol is observed to sensitize carfilzomib-induced apoptosis through the upregulation of SMAC, and downregulation of SIRT1, a positive modulator of survivin (170). Resveratrol also induced apoptosis in K562 cells through the activation of p38 and JNK, and the inhibition of ERK; it also increased caspase 3 cleavage as well as the expression of bim (171). In the treatment of multiple myeloma cells with bortezomib and gambogenic acid, a prenylated xanthone was observed to induce apoptosis via the activation of PARP cleavage, P53, Caspase-3 cleavage and Bax and inhibition of Bcl-2 expression (172). Curcumin also synergises with carfilzomib to significantly downregulate the nf-kb pathway (173). In a recent clinical study, Ramakrishna et al. showed that oral administration of up to 8 g of curcumin daily to MM patients is well tolerated and can decrease the paraprotein load, free light chains, bone turnover, and% plasma cell dyscrasia (174). Zaidi et al. reported similar activities of curcumin in a multiple relapsed MM patient on curcumin (175). quercetin and kaempferol derivatives have been shown to induce activation of caspase-3, -8 and -9, subsequent cleavage of PARP, and significantly suppressed XIAP, cIAP-1 and cIAP-2 in a dose-dependent manner along with the upregulation of proteins (Bax and Bad), and downregulation of anti-apoptotic proteins (Bcl-2 and Bcl-xL) and cytochrome *c* release (176).

These studies provide considerable evidence that polyphenols can induce apoptosis in hematological cancers, through the activation of death receptors, upregulation of pro-apoptotic proteins and induction of caspase 8, 3, and 9. Moreso, PI3K/Akt/mTOR pathway is an important pathway for the growth, survival and chemoresistance of leukemic cells. It is targeted especially in lymphoproliferative neoplasms (113, 177). Polyphenols can therefore be attractive candidates for this pathway in the management of hematological malignancies.

## Cell cycle

**FIGURE 5:**
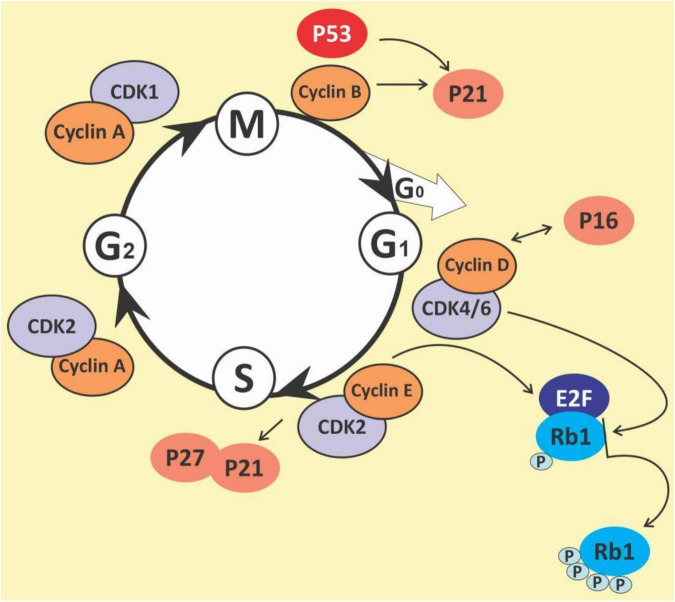
The cell cycle.

The cell cycle is a complex process that involves numerous regulatory proteins that direct the cell through a specific sequence of events culminating in mitosis and the production of two daughter cells. It is a fundamental step in the growth, development and maintenance of living things. It has two basic stages it passes through to divide and produce new cells. These are the interphase (the S phase, where cells duplicate their DNA contents through DNA replication; the G1 phase, where cells synthesize mRNA and proteins in preparation for mitosis; G2 phase, a period of rapid protein synthesis. It is a point in the G2 phase of the cell cycle where cells become arrested in response to DNA damage) (178, 179); M (Mitotic) phase, chromosome segregation and cell division take place at this phase (consists of prophase, metaphase, anaphase and telophase). The cell cycle is a closely-controlled process by a family of serine/threonine protein-dependent kinases known as cyclin-dependent kinases (CDKs) (180) (Figure 5). Their regulatory subunits are known as cyclins and are involved in the regulation of CDK’s activities. CDK activities are also regulated by endogenous CDK inhibitors. There are two families of CDK inhibitors, the inhibitor of cyclin-dependent kinase 4 (INK4) family and the CDK interacting protein/kinase inhibitory protein (Cip/Kip) family (181, 182). The INK4 family includes p15 (INK4b), p16 (INK4a), p18 (INK4c), and p19 (INK4d), whilst the Cip/Kip family includes p21 (Cip1/Waf1), p27 (Cip2), and p57 (Kip2) (183, 184). They play a role in the inhibition of the CDK-cyclin complexes, thereby halting the cell cycle progression. Some other proteins play a role in the cell cycle. These are proto-oncogenes and they fall into two categories: gain-of-function mutations in proto-oncogenes, which enhance cell growth and division; and loss-of-function mutations in tumor suppressor genes that inhibit unhindered cell growth and cell cycle checkpoint activation among other things (185). The loss of function mutations includes the p53 and retinoblastoma (Rb) protein (186) while the gain of function mutations include K-ras and Bcr-abl protein (187). The Rb family of proteins play a key role in the regulation of the cell cycle progression from the G1 to S phase. This function is achieved through the negative regulation of the E2F transcription factors and the binding to histone deacetylases and chromatin remodeling complexes. Mitogenic signaling leads to the activation of CDKs, especially CDK 4 and 6 which phosphorylates and inactivates Rb protein leading to E2F activation and its target genes (188). The p53 protein is an important element in cell cycle regulation and apoptosis. It is called the guardian of the genome because of its role in tumor initiation. It performs multiple regulatory functions by receiving information, modulating and relaying the information, and carrying out multiple downstream signals such as cellular senescence, cell metabolism, inflammation, autophagy, and other biological processes which control the survival and death of abnormal cells (189, 190). Mdm2 and MdmX are negative regulators of p53. Mdm2 promotes Lys ubiquitination at the C-terminus, targeting p53 for proteasomal degradation (191). Due to its central regulatory role in tumor development, it is known as a tumor suppressor protein (192, 193). The p53 protein is reported to be the most mutated gene in most human cancers with a frequency of about 50% (194), however, it has a low incidence in hematological malignancies (195, 196).

## Polyphenols and cell cycle arrest

The cell cycle has been observed as one key area for cancer cell proliferation. The cyclins and CDKs play an important role in the cell cycle and are known to be up or downregulated in several cancers including lymphomas and leukemias (197). Resistance to chemotherapy has been linked to the G0 phase of the cell cycle as well as the overexpression of some cyclins in cancers (198–200). Given the importance of the cyclins and CDKs for cell cycle control, these make attractive targets for chemotherapeutic intervention in hematological malignancies. Busa et al., reported that palbociclib, a breast cancer-approved CDK4/6 inhibitor suppressed AML in patient-derived Xenograft (201). Thus, G0/G1 phase and cyclin D1 are potential targets for the management of hematological malignancies (202, 203). Polyphenols and polyphenol-rich extracts have equally shown potential as cell cycle inhibitors (204, 205). Shih et al. showed that the polyphenol fraction of jelly fig (*Ficus awkeotsang* Makino) achenes caused G2/M cell cycle arrest in U937 cells (206). Resveratrol has been reported to arrest cell cycle progression in HL-60 leukemia cells by inducing the overexpression of cyclins A and E (207). Resveratrol has also been reported to inhibit cell cycle progression among other activities in acquired drug-resistant cancer cell lines including leukemia (208). Punicalagin, quercetin and delphinidin also induced G0/G1 and S phase cell cycle arrest in Jurkat, MOLT-3, HL-60, THP-1 and KG-1a leukemia cell lines (209, 210). Pomegranate juice has also been muted to exert some antileukemic effects; this was reported in a 44-year-old Caucasian man who was diagnosed with a T cell lymphoblastic lymphoma but had spontaneous remission without any chemotherapy treatment. The patient admitted to regularly drinking pomegranate juice, during the period after diagnosis. However, there was a tumor recurrence. Pomegranate juice extracts could be speculated to have caused the initial spontaneous remission (211). The pleiotropic molecule curcumin has been shown to induce G1 phase arrest in HL-60 cells and G2/M phase arrest in K562 cells (212), upregulate p21 and inhibit cyclin D1 in ML-2 and OCI-AML5 cells (213), and downregulation of cyclin D1, downregulation MDM2 and increase in p53 in multiple myeloma cell line (214). Quercetin, apigenin, emodin, rhein and *cis*-stilbene have all been shown to act synergistically with doxorubicin and etoposide to cause S and/or G_2_/M phase cell cycle arrest in lymphoid leukemia cell lines (215). 5-fluorouracil when combined with quercetin, apigenin and rhein caused a synergistic decrease in ATP levels, and induction of cell-cycle arrest in leukemia cell lines (216). The chalcone butein has also been shown to markedly downregulate the protein expression levels of CDK4, CDK6, cyclin D1, cyclin D2, cyclin E and phospho-pRb in HTLV-1-infected T cells, both *in vitro* and *in vivo* suggesting its therapeutic potentials in ATLL (217). It is thus evident that polyphenols are capable of both reducing CDKs, whilst increasing p53, resulting in cell cycle arrest and highlighting their therapeutic potential in preventing cell cycle progression and cell division in hematological malignancies.

## The kynurenine pathway

**FIGURE 6:**
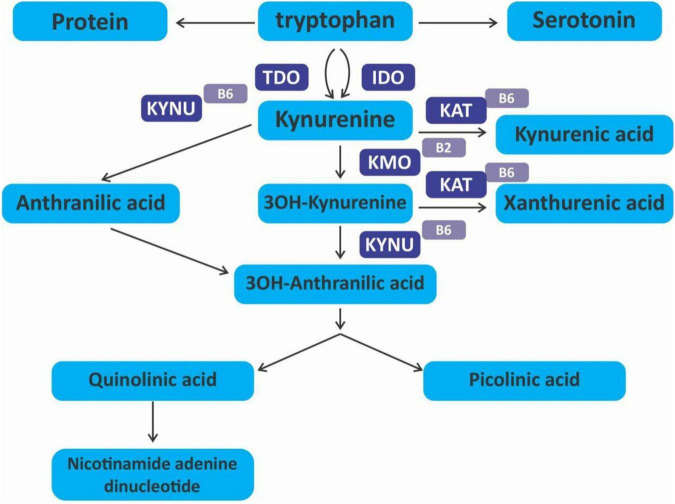
Kynurenine pathway.

**FIGURE 7:**
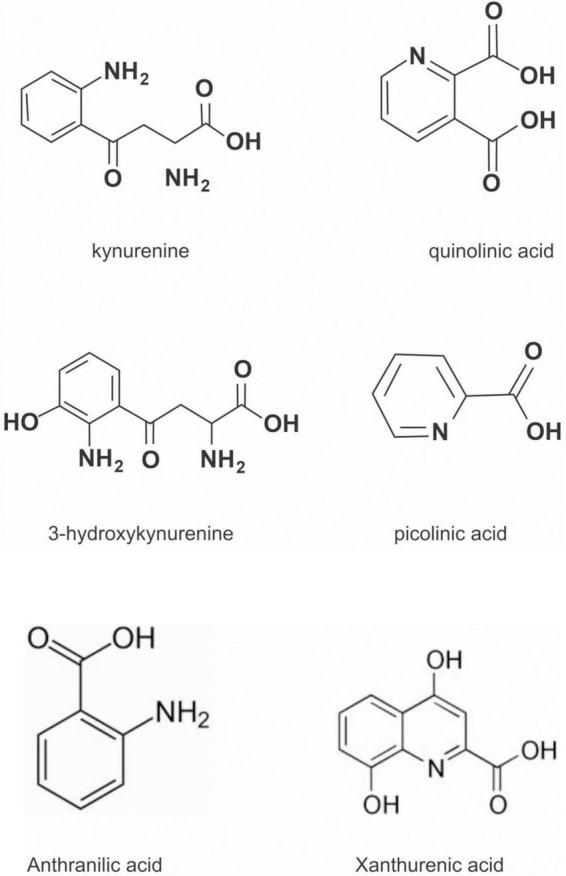
Cheemical structure of kynurenine pathway metabolites.

The kynurenine pathway (KP) is a metabolite pathway that is involved in generating cellular energy in the form of nicotinamide adenine dinucleotide (NAD+) (218). Tryptophan is the starting block of the pathway and 99% of it is catabolised in this pathway, if not incorporated into proteins via protein synthesis (Figure 6) (219). The conversion of tryptophan to kynurenine is mediated by either indoleamine 2,3-dioxygenase (IDO) or by tryptophan 2,3-dioxygenase (TDO) as rate-limiting enzymes. The KP is involved in the depletion of serum tryptophan and its conversion to biologically active metabolites. These metabolites include kynurenic acid, 3-hydroxykynurenine, anthranilic acid, xanthurenic acid, picolinic acid and quinolinic acid (Figure 7). These metabolites, along with the enzymes responsible for their production, have implications in a plethora of disease states. Chief among these enzymes are the rate-limiting enzymes that aid the conversion of tryptophan to kynurenine, indoleamine 2,3-dioxygenase (IDO) and tryptophan 2,3-dioxygenase (TDO). IDO is a heme-containing enzyme physiologically expressed in a number of tissues and cells. IDO is encoded by the IDO1 gene located on chromosome 8. IDO1is primarily regulated at the transcriptional level, and the regulatory proteins involved are (i) NF-KB (220), (ii) the aryl hydrocarbon receptor (AhR) (221, 222), and (iii) CTCF (223). Endogenous NO production can cause proteasomal degradation of IDO1 (224), but IFN-gamma can upregulate mRNA expression (225). IDO1 and its cognate tryptophan metabolites have been described to have immunomodulatory properties. Kynurenine can control T-cell immune responses especially through the generation of FoxP3^+^ T regulatory cells via AhR binding (226, 227); 3-hydroxykynurenine aids the depletion of CD4(+) T, CD8(+) T, B lymphocytes and induce the action of regulatory T cells (228); 3-Hydroxyanthranilic acid has immunomodulatory effects on macrophages and lymphocytes through the inhibition of PI3K/Akt/mTOR and NF-κB activation (229, 230) and inhibit Th1 and Th2 cells and increase the percentage of regulatory T cells; quinolinic acid is known to confer resistance to cancers (231); picolinic acid suppresses proliferation and metabolic activity of CD4 + T cells (232).

IDO1 activity has been associated with many diseases including hepatitis B infection (233), malaria (234), psychiatric disorders (235), atherosclerosis (236) as well as cancer and the immune escape often observed in tumors (237, 238). IDO1 was originally thought to be an anti-cancer molecule because of its ability to deplete the tryptophan needed for cell metabolism and growth. However, the immunosuppressive ability has shown it is more of a pro-cancer molecule. IDO1 is overexpressed in more than 50% of tumors (239) including hematological malignancies. Like in solid tumors, hematological malignancies are known to create an immunosuppressive environment to foster immunological tolerance of cancer cells. IDO1 has been described as one of the ways used for immunosuppression in several hematological tumors. While its mechanism is not well understood, increased IDO1 and kynurenine are associated with the inhibition of NK cell function (129, 240), activation of T regulatory cells (241); and recruitment and activation of myeloid-derived suppressor cells (MDSCs) (242). All these foster the immune escape of cancer cells. AML cells, but not normal hematopoietic stem cells (HSCs), have been shown to constitutively express IDO1 (243) which in turn causes an increase in circulating CD4 + CD25 + FOXP3 + t cells in AML patients. A recent systematic review by Wells et al. shows that IDO expression in AML is associated with poor prognosis (244) and measurement of IDO and its kynurenine metabolites may be incorporated into prospective prognostic algorithms (245). It also confers a poor prognosis in childhood AML (246, 247). In CLL, kynurenine-treated CLL cells are more resistant to the apoptotic effect of venetoclax, a bcl-2 inhibitor (248). While pro-inflammatory mediators such as tumor necrosis factor-alpha (TNF-α) can induce an increase in IDO activity by acting synergistically with IFN-γ (249), anti-inflammatory cytokines such as interleukin (IL)-10 inhibit IDO activity (250). There is an association between IDO1 expression and cyclooxygenase (COX)-2. Studies have shown that the COX-2 inhibitor celecoxib inhibits IDO-mediated immune tolerance through regulatory T cells as well as suppresses the Interferon-γ-Induced expression of indoleamine 2,3-dioxygenase (IDO) in human leukemia cell lines (251, 252). Thus, suggesting the use of COX-2 inhibitors as potential drugs to circumvent IDO1-mediated immune tolerance in AML. From previous studies, it is known that anti-inflammatory compounds like salicylic acid slow down Th-1 type immune response, slowing down tryptophan breakdown (253). Coffee extracts were also reported to prevent tryptophan breakdown, essentially preventing the effects of kynurenine and other metabolites (254). In recent studies, several flavonoids including baicalein, mangiferin, EGCG, curcumin, etc., have been reported to correct the Th17/Treg imbalance restoring immunocompetence of effector T-cells (255).

The effect of IDO1 and kynurenine metabolites cannot be understated, especially their role in tumor immunology. Preclinical studies in a mouse model show that IDO inhibitor, DL-methyltryptophan suppresses tumor growth and peritoneal dissemination, and increases the efficacy of chemotherapeutic agents (256). Polyphenols are able to regulate these actions and thus can act as an adjunct in cancer immunotherapy (257, 258). Resveratrol has been shown to regulate IDO1 in a JAK/STAT1- and PKCδ-dependent manner (259). Curcumin also inhibited IDO1 in a JAK/STAT1- and PKCδ-dependent manner and also reversed IDO-mediated suppression of T-cell responses (260). However, curcumin does downregulate IDO expression via a COX-2/PGE2-dependant pathway (261). EGCG has been shown to inhibit the transcriptional activities of IDO promoters, IFN-stimulated response element and IFN-γ activation sequence, activated by STAT1 phosphorylation as well as the enzymatic activity of IDO1 (262). This is in contrast to flavones such as apigenin, baicalein, chrysin, and wogonin which inhibit the enzymatic activity of IDO-1 but not mRNA expression (263). Furthermore, EGCG inhibited the expression of COX-2 and the production of Prostaglandin E(2) (264).

Studies have suggested that IDO inhibition could be used therapeutically in cancer treatment especially AML (265). One study showed the use of the IDO inhibitor, 1-methyl tryptophan (1MT) with adriamycin in AML caused significant inhibition of blast cell proliferation and a significant increase in lymphocyte counts when used alone (266). Nakamura et al. also showed that a combination of 1MT and cyclophosphamide is an effective treatment for IDO-positive lymphoma in a model mouse by reducing Tregs and breaking tumor tolerance (267). However, failure of phase III clinical trial (ECHO-301/KN-252) where Epacadostat an IDO inhibitor in combination with anti-PD-1 antibody pembrolizumab was used in metastatic melanoma patients did not demonstrate improved progression-free survival and OS and thus terminated early (268, 269) have pushed for a re-think on the clinical benefits of IDO inhibitors in cancer. However, indoximod, another IDO inhibitor in phase 1 clinical trial was shown to be well-tolerated and induced a high rate of complete remission with MRD-negativity in newly diagnosed AML patients (270). In phase II clinical trial of patients with advanced melanoma, indoximod in combination with pembrolizumab was well tolerated and showed antitumor efficacy that was worth further evaluation (271). A phase II clinical trial of indoximod with chemotherapy and radiotherapy in pediatric cancer patients is currently ongoing (NCT04049669). Other targets of the kynurenine pathway are being muted for cancer immunotherapy such as TDO inhibitors (272) and AhR inhibitors (273). Another proposed option is the use of COX2 inhibitors since COX2 enhance the expression of IDO1 in tumors (274, 275). Polyphenols do inhibit COX-2 in cancer cells (276, 277) Celecoxib have also been shown to exert antineoplastic activity in AML cell lines (278) as well as in CML cell line (279). Polyphenols can be used as immunomodulatory agents in combination with some established therapies to attenuate the kynurenine pathway or enhance cellular immunity in hematological malignancies. In a clinical study of elderly AML patients, green tea was reported to exert an immunomodulatory effect in combination with low-dose cytarabine (280). Various studies have shown that the expression of IDO1 in AML portends a poor prognosis (244, 281, 282). Targeting hematological malignancies with IDO1 or COX2 polyphenolic inhibitors may be another therapeutic option.

## Polyphenols in hematological malignancies: Clinical studies

Preclinical studies have shown the efficacy of various polyphenols such as curcumin, apigenin, EGCG, quercetin, resveratrol, etc., in cancer. They have been studied extensively both *in vitro* and *in vivo* by various groups and found to have good activity against different types of cancer. However, clinical studies using natural products including polyphenols are still in infancy and are often targeted at improving the efficacy of standard chemotherapy and also reducing the adverse reactions from chemotherapy. Most clinical trials are however, targeted at solid tumors (33). This may be because of the successes recorded in the non-phytochemical-based therapies especially the immune-based ones (283). The Food and Drug Administration (FDA) from 2011 to 2021 approved 52 new drug registrations for hematological malignancies; 29 of them were for small molecule drugs and 23 of them were for macromolecules (284). Flavopiridol (Alvocidib) a plant-derived semisynthetic flavone that acts as a cyclin-dependent kinase inhibitor was given an orphan drug designation in CLL, but subsequent studies showed it has significant activity against CLL as well as significant toxicities (285). Some other phase II studies as a combination therapy in AML showed it had higher rates of complete remission and a similar toxicity profile when compared to chemotherapy-only treatment (286, 287). In a recent phase II trial of three novel regimens against AML, the flavopiridol combination therapy regimen had a higher response rate than the other two regimens showing it could be pursued for further clinical development (288). Recently, a novel flavopiridol formulation was developed which showed improved pharmacokinetics and efficacy against AML both *in vitro* and *in vivo* (289). This shows the potential of polyphenols in leukemia management.

In multiple myeloma, curcumin and curcumin analogs in clinical trials were reported to have significant activity and clinical response (290). In a cohort study of 52,000 adults followed for 13 years, the consumption of green tea was observed to be inversely proportional to the risk of total hematological malignancies especially AML (291). The use of polyphenols in the management of light chain amyloidosis is considered with some interest. A case of improvement in cardiac symptoms of AL amyloidosis in a patient purposely drinking high amounts of green tea have been reported (292, 293).

Some clinical trials on these are still on (NCT01511263, NCT02015312) (294). Similarly, in a phase I and phase II clinical trial with CLL patients (Rai stage 0 to II), green tea extracts with doses ranging from 400 to 2,000 mg showed a good tolerance, as well as a decline in both the absolute lymphocyte count and in lymphadenopathy (295–297). In a cohort of 11 patients with various indolent lymphomas (CLL, follicular lymphoma (FL), Waldenstrom macroglobulinemia (WM), monoclonal gammopathy of undetermined significance (MGUS), and splenic Marginal zone lymphoma (MZL) who were given two bags of green tea daily and followed up, there was a clinical response with improvement in biomarkers and lymphadenopathy (298). In the IDEAL trial, the use of caloric restriction and increased intake of proteins and polyphenol-rich diets boosted the effectiveness of chemotherapy in acute leukemia patients (299). PIM1 kinase positive CLL patients were given quercetin therapy (500 mg twice daily) in a study; clinical response along with zero toxicity were noted (300). In a recent phase I trial, combretastatin a stilbene from the African Bushwillow Combretum caffrum was added to cytarabine in relapsed/refractory AML and it showed an overall response rate of 19% with a significantly longer overall survival in those that achieved a complete remission (301).

Despite the various drawbacks, it is evident that polyphenols are safe for human clinical trials and can serve some purpose in the management of hematological malignancies. These compounds should be considered serious candidates and efforts should be intensified to set up a well-planned clinical trial to consider them for approval.

## Delivery system for polyphenols

The roles polyphenols play in cell regulation and cancer formation cannot be understated. They along with other phytochemicals are usually the mainstay of traditional herbal medicine. Wherein polyphenols are an important source of possible therapeutic agents, their major drawbacks are their bioavailabilities and pharmacokinetics. Oral administration of polyphenols has varying absorption potential according to their chemical nature. The presence of functional groups can also affect polyphenol absorption. Overcoming these challenges is needed to get polyphenols into the clinics. One of the ways attempted to overcome this challenge is the synthesis of polyphenol analogs. Analogs have been shown to improve compound stability and their bioavailability (302). The curcumin analog EF24 and EF31have been shown to have increased bioavailability (303) and with good anticancer activity (304, 305). Another set of curcumin analogs GO-Y078 and GO-Y030 were discovered to be 7 to 12-fold more potent growth inhibitors for myeloma cells, and 6- to 15-fold more powerful suppressors of IRF4, JAK/STAT3, PI3K/AKT, and NF-κB pathways than curcumin (306). EGCG synthetic analogs are also known to possess anticancer activities through several mechanisms (307). Thus, polyphenol analogs are one of the ways to improve their bioavailability and efficacy.

Nanotechnology is a promising tool to enhance the efficacy and delivery of drugs. The use of nanotechnology is expected to solve the problem of bioavailability and bioactivities of polyphenols by reducing particle size as a drug. A curcumin chitosan nanoparticle developed was found to have a tenfold increase of curcumin over native curcumin (308). A number of FDA-approved nanodrugs are on the market including vyxeos liposomal used in the management of AML and marqibo for the management of ALL (309). Thus, the aspect of the use of polyphenol-laden nanoformulations as anticancer therapies is a possibility. Curcumin nanodisks have been reported to induce apoptosis in mantle cell lymphoma and with improved bioavailability (310). Resveratrol nanoformulations in combination with standard chemotherapies have been tested across various cancers both *in vitro* and *in vivo* with good bioavailability and bioactivity reported (311, 312). Thus, resveratrol-based nanoformulations are being seen as a viable option in cancer treatment (313). A nano-drug delivery system with folic acid-functionalized EGCG showed good bioavailability and enhanced toxicity to ovarian cancer cells both *in vitro* and *in vivo* (314) showing potential as a treatment option.

Conjugated antibodies for cancer therapy are a well-developed strategy. They are composed of a monoclonal antibody tethered to a cytotoxic drug (known as the payload) via a chemical linker. They target the specificity of a monoclonal antibody to reach target antigens expressed on cancer cells for the delivery of a potent cytotoxic payload. To date, nine conjugated antibodies have been approved by the FDA and more than 80 conjugated antibodies are under clinical development worldwide (315). Examples include inotuzumab ozogamicin a recombinant humanized IgG4 conjugated antibody used in the management of B cell precursor ALL (316). Ozogamicin is the drug conjugate, a natural product from the class of calicheamicins (a class of enediyne antitumor antibiotics derived from the bacterium Micromonospora echinospora). Polyphenol antibody conjugate is also a strategy to deliver drugs to cancer cells. Polyphenol antibody conjugate is also known to improve bioavailability as well as efficacy. Nirachonkul et al. showed that anti-CD123-curcumin-loaded PLGA/poloxamer nanoparticles (anti-CD123-Cur-NPs) exhibited more cytotoxicity than curcumin-loaded PLGA/poloxamer nanoparticles (Cur-NPs) in leukemia stem cells (317). In another experiment, antibody-coupled curcumin was 230-fold more effective in eliminating B16F10 melanoma cells *in vitro*, and *in vivo* compared to curcumin alone, and also more efficacious than antibodies against the melanoma surface antigen Muc18 (318). These results show that the conjugation of some polyphenols can be efficacious against some hematological malignancies and can be explored further in clinical trials.

Hybrid combinations, a term coined by Wagner and Efferth in 2017 can be described as a combination of synthetic drugs with chemically defined constituents from plants (secondary metabolites) aiming to increase the pharmacological activity of the formulation and simultaneously reduce the toxic side-effects of the drugs (319). The synergy created by the hybrid combinations increases chemotherapy cytotoxicity and overcome resistance through their multi-target actions (320). Quite many hybrid combinations have been described for various cancer therapies (321). The combination of a chemotherapy formulation of cysteamine-modified cadmium tellurium (Cys-CdTe) quantum dots coloaded with daunorubicin and gambogic acid (GA) nanoparticles displayed a dose-dependent antiproliferative activity on multidrug-resistant lymphoma Raji/DNR cells *in vitro* and *in vivo* (322). Also, the curcumin-thalidomide hybrid combination was tested on MM1S, RPMI18226 and U266 human multiple myeloma (MM) cells and observed to generate higher levels of ROS after treatment and other biological activities compared to curcumin alone (323). Similarly some polyphenols especially apigenin have been noted to enhance the efficacy of alkylating agents in leukemia cell lines (324). Hybrid combinations have shown potential, and have even led to the creation of integrative oncology programs in some universities (325).

## Future perspectives

This review have shown the potentials of polyphenols and their viability as either alternatives or complimentary options in the management of hematological malignancies. More attention are being focused on polyphenols in recent times probably because of their dexterity and pleiotropic effects. This interest can be seen in the number of recent research articles published over the past two decades. For example, between 1966 and 2004 only four scientific studies were published on gambogic acid, but since 2004 more than 370 reports for its general medicinal applications have been published of which about 260 are on cancers (326). This increased interest have led to additional studies of gambogic acid as a combination therapy with bortezomib to determine efficacy in multiple myeloma (327, 328). A phase II clinical trial also noted its dosage and safety profile in malignant tumors (329), and the fact that it does not cause bone marrow suppression was a plus (330). Unfortunately, like other polyphenols bioavailability is low, thus limiting its clinical potentials (331). In order to improve its clinical efficacy, several delivery systems such as micelles, nanoparticles and structural modifications are being deployed for greater availability (332, 333).

Lately, targeting the immune system have gained much grounds in the management of cancers in general and immune-based therapies are readily available for cancer treatment. The immunomodulatory activities of polyphenols are well documented (334, 335), and they are seen as possible immunoadjuvants (257). For example, apigenin have been shown to reduce the expression of PD-L1 in melanoma cells (336) as well as in K-ras mutant lung carcinoma *in vivo* (337). A curcumin analog bisdemethoxycurcumin in combination with an anti-PD-L1 antibody was able to cause an increase in CD8 + T cells as well as reduce PD-1 expression in an *in vivo* mouse model of bladder cancer (338).

Given the complexity of hematological malignancies, the use of combination therapies that target multiple signaling pathways is a standard management practice. Polyphenol fits in well for such combination therapies. However, improving their bioavailability is necessary to achieve their full potentials. It is hoped that one or more of the polyphenols will pass through phase III clinical trials sucessfully and find its way to the clinics.

## Conclusion

Taking into account the advances in the areas of pharmacotherapy and hematological cancer research, it is evident that polyphenols have an important role to play hematological malignancies which I propose can come in the form of combination chemotherapy (339) or maintenance therapy (340). However, before polyphenol-based cancer therapies can be deployed to the clinics, further pre-clinical studies and clinical trials would be needed to be done to validate their use. This will ensure safety and standards in the clinical settings.

## Author contributions

The author confirms being the sole contributor of this work and has approved it for publication.

## References

[1] Sung H Ferlay J Siegel RL Laversanne M Soerjomataram I Jemal A Global cancer statistics 2020: globocan estimates of incidence and mortality worldwide for 36 cancers in 185 Countries. *CA Cancer J Clin.* (2021) 71:209–49. 10.3322/caac.21660 33538338

[2] Swerdlow SH Campo E Harris NL Jaffe ES Pileri SA Stein H WHO Classification of Tumours of Haematopoietic and Lymphoid Tissues. In: Bosman FT Jaffe ES Lakhani SR Ohgaki H editors. *World Health Organization Classification of Tumours.* Lyon: IARC (2017).

[3] Bray F Ferlay J Soerjomataram I Siegel RL Torre LA Jemal A. Global cancer statistics 2018: GLOBOCAN estimates of incidence and mortality worldwide for 36 cancers in 185 countries. *CA Cancer J Clin.* (2018) 68:394–424. 10.3322/caac.21492 30207593

[4] Dong Y Shi O Zeng Q Lu X Wang W Li Y Leukemia incidence trends at the global, regional, and national level between 1990 and 2017. *Exp Hematol Oncol.* (2020) 9:14. 10.1186/s40164-020-00170-6 32577323 PMC7304189

[5] Gibson TM Morton LM Shiels MS Clarke CA Engels EA. Risk of non-Hodgkin lymphoma subtypes in HIV-infected people during the HAART era: a population-based study. *AIDS.* (2014) 28:2313–8. 10.1097/QAD.0000000000000428 25111081 PMC4260326

[6] Kieri O Marrone G Sönnerborg A Nowak P. Incidence, treatment, and outcome of HIV-associated hematologic malignancies in people living with HIV in Sweden. *AIDS Res Hum Retroviruses.* (2022) 38:135–42. 10.1089/AID.2021.0020 34652958

[7] Barnes JI Divi V Begaye A Wong R Coutre S Owens DK Cost-effectiveness of ibrutinib as first-line therapy for chronic lymphocytic leukemia in older adults without deletion 17p. *Blood Adv.* (2018) 2:1946–56. 10.1182/bloodadvances.2017015461 30097461 PMC6093732

[8] O’Brien SM Lamanna N Kipps TJ Flinn I Zelenetz AD Burger JA A phase 2 study of idelalisib plus rituximab in treatment-naïve older patients with chronic lymphocytic leukemia. *Blood.* (2015) 126:2686–94 10.1182/blood-2015-03-630947 26472751 PMC4732760

[9] Byrd JC Woyach JA Furman RR tin P O’Brien S Brown JR Acalabrutinib in treatment-naive chronic lymphocytic leukemia. *Blood.* (2021) 137:3327–38. 10.1182/blood.2020009617 33786588 PMC8670015

[10] Tam CS O’Brien S Wierda W Kantarjian H Wen S Do KA Long-term results of the fludarabine, cyclophosphamide, and rituximab regimen as initial therapy of chronic lymphocytic leukemia. *Blood.* (2008) 112:975–80. 10.1182/blood-2008-02-140582 18411418 PMC3952498

[11] Gupta SC Patchva S Aggarwal BB. Therapeutic roles of curcumin: lessons learned from clinical trials. *AAPS J.* (2013) 15:195–218. 10.1208/s12248-012-9432-8 23143785 PMC3535097

[12] Niedzwiecki A Roomi MW Kalinovsky T Rath M. Anticancer efficacy of polyphenols and their combinations. *Nutrients.* (2016) 8:552 10.3390/nu8090552 PMC5037537 27618095

[13] Weng JK Philippe RN Noel JP. The rise of chemodiversity in plants. *Science.* (2012) 336:1667–70. 10.1126/science.1217411 22745420

[14] Zhou Y Zheng J Li Y Xu DP Li S Chen YM Natural polyphenols for prevention and treatment of cancer. *Nutrients.* (2016) 8:515. 10.3390/nu8080515 27556486 PMC4997428

[15] Briguglio G Costa C Pollicino M Giambò F Catania S Fenga C. Polyphenols in cancer prevention: new insights (Review). *Int J Funct Nutr.* (2020) 1:9. 10.3892/ijfn.2020.9

[16] Manach C Scalbert A Morand C Rémésy C Jiménez L. Polyphenols: food sources and bioavailability. *Am J Clin Nutr.* (2004) 79:727–47. 10.1093/ajcn/79.5.727 15113710

[17] Speer H D’Cunha NM Botek M McKune AJ Sergi D Georgousopoulou E The effects of dietary polyphenols on circulating cardiovascular disease biokers and iron status: a systematic review. *Nutr Metab Insights.* (2019) 12:1178638819882739. 10.1177/1178638819882739 31673228 PMC6804354

[18] Alotaibi BS Ijaz M Buabeid M Kharaba ZJ Yaseen HS Murtaza G. Therapeutic effects and safe uses of plant-derived polyphenolic compounds in cardiovascular diseases: a review. *Drug Des Devel Ther.* (2021) 15:4713–32. 10.2147/DDDT.S327238 34848944 PMC8619826

[19] Singh A Yau YF Leung KS El-Nezami H Lee JC-Y. Interaction of polyphenols as antioxidant and anti-inflammatory compounds in brain–liver–gut axis. *Antioxidants.* (2020) 9:669. 10.3390/antiox9080669 32722619 PMC7465954

[20] Sharma A Kaur M Katnoria JK Nagpal AK. Polyphenols in food: cancer prevention and apoptosis induction. *Curr Med Chem.* (2018) 25:4740–57. 10.2174/0929867324666171006144208 28990504

[21] Kumar N Goel N. Phenolic acids: natural versatile molecules with promising therapeutic applications. *Biotechnol Rep.* (2019) 24:e00370. 10.1016/j.btre.2019.e00370 31516850 PMC6734135

[22] Ghosh S Chisti Y Banerjee UC. Production of shikimic acid. *Biotechnol Adv.* (2012) 30:1425–31. 10.1016/j.biotechadv.2012.03.001 22445787

[23] Martínez JA Bolívar F Escalante A. Shikimic acid production in *escherichia coli*: from classical metabolic engineering strategies to omics applied to improve its production. *Front Bioeng Biotechnol.* (2015) 3:145. 10.3389/fbioe.2015.00145 26442259 PMC4585142

[24] Huccetogullari D Luo ZW Lee SY. Metabolic engineering of microorganisms for production of aromatic compounds. *Microb Cell Fact.* (2019) 18:41. 10.1186/s12934-019-1090-4 30808357 PMC6390333

[25] Averesch NJH Krömer JO. Metabolic engineering of the shikimate pathway for production of aromatics and derived compounds-present and future strain construction strategies. *Front Bioeng Biotechnol.* (2018) 6:32. 10.3389/fbioe.2018.00032 29632862 PMC5879953

[26] Tzin V Galili G. Amino acids biosynthesis pathways in plants. *Molecular Plant.* (2010) 3:956–72. 10.1093/mp/ssq048 20817774

[27] Panche AN Diwan AD Chandra SR. Flavonoids: an overview. *J Nutr Sci.* (2016) 5:e47 10.1017/jns.2016.41 PMC5465813 28620474

[28] Samanta A Das G Das S. Roles of flavonoids in plants. *Int J Pharm Sci Tech.* (2011) 6:12–35.

[29] Liu J Wang X Yong H Kan J Jin C. Recent advances in flavonoid-grafted polysaccharides: synthesis, structural characterization, bioactivities and potential applications. *Int J Biol Macromol.* (2018) 116:1011–25. 10.1016/j.ijbiomac.2018.05.149 29800657

[30] Farias S Da Costa K Mertins J. Analysis of conformational, structural, magnetic, and electronic properties related to antioxidant activity: revisiting flavan, anthocyanidin, flavanone, flavonol, isoflavone, flavone, and flavan-3-ol. *ACS Omega.* (2021) 6:8908–18. 10.1021/acsomega.0c06156 33842761 PMC8028018

[31] Slámová K Kapešová J Valentová K. “Sweet Flavonoids”: glycosidase-Catalyzed Modifications. *Int J Mol Sci.* (2018) 19:2126. 10.3390/ijms19072126 30037103 PMC6073497

[32] Xiao J. Dietary flavonoid aglycones and their glycosides: which show better biological significance? *Crit Rev Food Sci Nutr.* (2017) 57:1874–905. 10.1080/10408398.2015.1032400 26176651

[33] Cháirez-Ramírez MH de la Cruz-López KG García-Carrancá A. Polyphenols as antitumor agents targeting key players in cancer-driving signaling pathways. *Front Pharmacol.* (2021) 12:710304. 10.3389/fphar.2021.710304 34744708 PMC8565650

[34] Jiang N Doseff AI Grotewold E. Flavones: from biosynthesis to health benefits. *Plants (Basel).* (2016) 5:27. 10.3390/plants5020027 27338492 PMC4931407

[35] Del Valle JC Buide ML Whittall JB Valladares F Narbona E. UV radiation increases phenolic compound protection but decreases reproduction in Silene littorea. *PLoS One.* (2020) 15:e0231611. 10.1371/journal.pone.0231611 32555603 PMC7302690

[36] Catarino MD Alves-Silva JM Pereira OR Cardoso SM. Antioxidant capacities of flavones and benefits in oxidative-stress related diseases. *Curr Top Med Chem.* (2015) 15:105–19. 25547095

[37] Hooper AM Hassanali A Chamberlain K Khan Z Pickett JA. New genetic opportunities from legume intercrops for controlling *Striga* spp. Parasitic weeds. *Pest Manag Sci.* (2009) 65:546–52. 10.1002/ps.1731 19266493

[38] Lan W Lu F Regner M Zhu Y Rencoret J Ralph SA Tricin, a flavonoid monomer in monocot lignification. *Plant Physiol.* (2015) 167:1284–95. 10.1104/pp.114.253757 25667313 PMC4378158

[39] Ahuja I Kissen R Bones AM. Phytoalexins in defense against pathogens. *Trends Plant Sci.* (2012) 17:73–90. 10.1016/j.tplants.2011.11.002 22209038

[40] Hostetler GL Ralston RA Schwartz SJ. Flavones: food sources, bioavailability, metabolism, and bioactivity. *Adv Nutr.* (2017) 8:423–35. 10.3945/an.116.012948 28507008 PMC5421117

[41] Mahbub AA Le Maitre CL Haywood-Small SL McDougall GJ Cross NA Jordan-Mahy N Differential effects of polyphenols on proliferation and apoptosis in human myeloid and lymphoid leukemia cell lines. *Anticancer Agents Med Chem.* (2013) 13:1601–13 10.2174/18715206113139990303 23796248 PMC3873039

[42] Budhraja A Gao N Zhang Z Son YO Cheng S Wang X Apigenin induces apoptosis in human leukemia cells and exhibits anti-leukemic activity *in vivo*. *Mol Cancer Ther.* (2012) 11:132–42. 10.1158/1535-7163.MCT-11-0343 22084167 PMC4430727

[43] Mahbub AA Le Maitre CL Cross NA Jordan-Mahy N. The effect of apigenin and chemotherapy combination treatments on apoptosis-related genes and proteins in acute leukaemia cell lines. *Sci Rep.* (2022) 12:8858 10.1038/s41598-022-11441-z 35614109 PMC9132959

[44] Zhang Q Zhao X Qiu H. Flavones and Flavonols: Phytochemistry and Biochemistry. In: Ramawat K Mérillon JM editors. *Natural Products.* Berlin: Springer (2013). p. 1821–47. 10.1007/978-3-642-22144-6_60

[45] Crozier A Burns J Aziz AA Stewart AJ Rabiasz HS Jenkins GI Antioxidant flavonols from fruits, vegetables and beverages: measurements and bioavailability. *Biol Res.* (2000) 33:79–88. 10.4067/s0716-97602000000200007 15693274

[46] Kothari D Lee W-D Kim S-K. *Allium* flavonols: health benefits, molecular targets, and bioavailability. *Antioxidants.* (2020) 9:888. 10.3390/antiox9090888 32961762 PMC7555649

[47] Torres-Villarreal D Camacho A Castro H Ortiz-Lopez R de la Garza AL. Anti-obesity effects of kaempferol by inhibiting adipogenesis and increasing lipolysis in 3T3-L1 cells. *J Physiol Biochem.* (2019) 75:83–8. 10.1007/s13105-018-0659-4 30539499

[48] Yen SC Chen LC Huang HL Ngo ST Wu YW Lin TE Investigation of selected flavonoid derivatives as potent FLT3 inhibitors for the potential treatment of acute myeloid leukemia. *J Nat Prod.* (2021) 22:1–10. 10.1021/acs.jnatprod.0c00589 33393294

[49] Shi H Li XY Chen Y Zhang X Wu Y Wang ZX Quercetin induces apoptosis via downregulation of vascular endothelial growth factor/akt signaling pathway in acute myeloid leukemia cells. Front pharmacol. 2020;11:534171. Erratum. *Front Pharmacol.* (2021) 11:640750. 10.3389/fphar.2020.534171 33362534 PMC7758733

[50] Moradzadeh M Tabarraei A Sadeghnia HR Ghorbani A Mohamadkhani A Erfanian S Kaempferol increases apoptosis in human acute promyelocytic leukemia cells and inhibits multidrug resistance genes. *J Cell Biochem.* (2018) 119:2288–97 28865123 10.1002/jcb.26391

[51] Yen SC Wu YW Huang CC Chao MW Tu HJ Chen LC O-methylated flavonol as a multi-kinase inhibitor of leukemogenic kinases exhibits a potential treatment for acute myeloid leukemia. *Phytomedicine.* (2022) 100:154061. 10.1016/j.phymed.2022.154061 35364561

[52] Del Rio D Rodriguez-Mateos A Spencer JP Tognolini M Borges G Crozier A. Dietary (poly)phenolics in human health: structures, bioavailability, and evidence of protective effects against chronic diseases. *Antioxid Redox Signal.* (2013) 18:1818–92. 10.1089/ars.2012.4581 22794138 PMC3619154

[53] Luo Y Jian Y Liu Y Jiang S Muhammad D Wang W. Flavanols from nature: a phytochemistry and biological activity review. *Molecules.* (2022) 27:719 10.3390/molecules27030719 PMC8838462 35163984

[54] Haytowitz DB Wu X Bhagwat S. *USDA Database for the Flavonoid Content of Selected Foods Release 3.3 Prepared by.* Washington, DC: U.S. Department of Agriculture, Agricultural Research Service (2018).

[55] Zhang L Chen QS Xu PP Qian Y Wang AH Xiao D Catechins induced acute promyelocytic leukemia cell apoptosis and triggered PML-RARα oncoprotein degradation. *J Hematol Oncol.* (2014) 7:75. 10.1186/s13045-014-0075-3 25270015 PMC4197244

[56] Zhou CG Hui LM Luo JM. Epigallocatechin gallate inhibits the proliferation and induces apoptosis of multiple myeloma cells via inactivating EZH2. *Eur Rev Med Pharmacol Sci.* (2018) 22:2093–8. 10.26355/eurrev_201804_14742 29687868

[57] Della Via FI Shiraishi RN Santos I. Ferro KP Salazar-Terreros MJ Franchi GC (–)-Epigallocatechin-3-gallate induces apoptosis and differentiation in leukaemia by targeting reactive oxygen species and PIN1. *Sci Rep.* (2021) 11:9103. 10.1038/s41598-021-88478-z 33907248 PMC8079435

[58] Crozier A Jaganath IB Clifford MN. Dietary phenolics: chemistry, bioavailability and effects on health. *Nat Prod Rep.* (2009) 26:1001–43. 10.1039/b802662a 19636448

[59] Ramesh P Jagadeesan R Sekaran S Dhanasekaran A Vimalraj S. Flavonoids: classification, function, and molecular mechanisms involved in bone remodelling. *Front Endocrinol.* (2021) 23:779638. 10.3389/fendo.2021.779638 34887836 PMC8649804

[60] Al-Khayri JM Sahana GR Nagella P Joseph BV Alessa FM Al-Mssallem MQ. Flavonoids as potential anti-inflammatory molecules: a review. *Molecules.* (2022) 27:2901. 10.3390/molecules27092901 35566252 PMC9100260

[61] Vetrivel P Kim SM Saralamma VVG Ha SE Kim EH Min TS Function of flavonoids on different types of programmed cell death and its mechanism: a review. *J Biomed Res.* (2019) 33:363–70. 10.7555/JBR.33.20180126

[62] Křížová L Dadáková K Kašparovská J Kašparovský T. Isoflavones. *Molecules.* (2019) 24:1076. 10.3390/molecules24061076 30893792 PMC6470817

[63] Jeandet P. Phytoalexins: current progress and future prospects. *Molecules.* (2015) 20:2770–4. 10.3390/molecules20022770

[64] Szeja W Grynkiewicz G Rusin A. Isoflavones, their glycosides and glycoconjugates. Synthesis and biological activity. *Curr Org Chem.* (2017) 21:218–35. 10.2174/1385272820666160928120822 28553156 PMC5427819

[65] Tsuchihashi R Sakamoto S Kodera M Nohara T Kinjo J. Microbial metabolism of soy isoflavones by human intestinal bacterial strains. *J Nat Med.* (2008) 62:456–60. 10.1007/s11418-008-0271-y 18648905

[66] Thrane M Paulsen PV Orcutt MW Krieger TM. Soy Protein: Impacts, Production, and Applications. In: Nadathur SR Wanasundara JPD Scanlin L editors. *Sustainable Protein Sources.* (Chap. 2), Cambridge: Academic Press (2017). p. 23–45. 10.1016/B978-0-12-802778-3.00002-0

[67] Pandey A Misra P Khan MP Swarnkar G Tewari MC Bhambhani S Co-expression of Arabidopsis transcription factor, AtMYB12, and soybean isoflavone synthase, GmIFS1, genes in tobacco leads to enhanced biosynthesis of isoflavones and flavonols resulting in osteoprotective activity. *Plant Biotechnol J.* (2014) 12:69–80 10.1111/pbi.12118 24102754

[68] Ma L Liu G Ding M Zong G Hu FB Willett WC Isoflavone intake and the risk of coronary heart disease in US men and women: results from 3 prospective cohort studies. *Circulation.* (2020) 141:1127–37 10.1161/CIRCULATIONAHA.119.041306 32200662 PMC7138725

[69] Tuli HS Tuorkey MJ Thakral F Sak K Ku M Sharma AK Molecular mechanisms of action of genistein in cancer: recent advances. *Front Pharmacol.* (2019) 10:1336. 10.3389/fphar.2019.01336 31866857 PMC6910185

[70] Kim SH Kim CW Jeon SY Go RE Hwang KA Choi KC. Chemopreventive and chemotherapeutic effects of genistein, a soy isoflavone, upon cancer development and progression in preclinical animal models. *Lab Anim Res.* (2014) 30:143–50. 10.5625/lar.2014.30.4.143 25628724 PMC4306701

[71] Boutas I Kontogeorgi A Dimitrakakis C Kalantaridou SN. Soy isoflavones and breast cancer risk: a meta-analysis. *In Vivo.* (2022) 36:556–62. 10.21873/invivo.12737 35241506 PMC8931889

[72] Chan KKL Siu MKY Jiang YX Wang JJ Leung THY Ngan HYS. Estrogen receptor modulators genistein, daidzein and ERB-041 inhibit cell migration, invasion, proliferation and sphere formation via modulation of FAK and PI3K/AKT signaling in ovarian cancer. *Cancer Cell Int.* (2018) 18:65. 10.1186/s12935-018-0559-2 29743815 PMC5930957

[73] Narasimhan K Lee YM Lim TK Port SA Han JH Chen CS Lin Q Genistein exerts anti-leukemic effects on genetically different acute myeloid leukemia cell lines by inhibiting protein synthesis and cell proliferation while inducing apoptosis – molecular insights from an iTRAQ™ quantitative proteomics study. *Oncoscience.* (2015) 2:111–24 10.18632/oncoscience.120 25859554 PMC4381704

[74] Yakimchuk K Revanna BC Huang D Inzunza J Okret S. Suppression of lymphoma growth by the xenoestrogens bisphenol A and genistein. *Endocr Connect.* (2018) 7:1472–9. 10.1530/EC-18-0459 30496125 PMC6300865

[75] Xie J Wang J Zhu B. Genistein inhibits the proliferation of human multiple myeloma cells through suppression of nuclear factor-kB and upregulation of microRNA-29b. *Mol Med Rep.* (2016) 13:1627–32. 10.3892/mmr.2015.4740 26718793

[76] Pereira DM Valentão P Pereira JA Andrade PB. Phenolics: from chemistry to biology. *Molecules.* (2009) 14:2202–11. 10.3390/molecules14062202

[77] El-Seedi HR El-Said AMA Khalifa SAM Göransson U Bohlin L Borg-Karlson A-K Biosynthesis, natural sources, dietary intake, pharmacokinetic properties, and biological activities of hydroxycinnamic acids. *J Agric Food Chem.* (2012) 60:10877–95. 10.1021/jf301807g 22931195

[78] Alam MA Subhan N Hossain H Hossain M Reza HM Rahman MM Hydroxycinnamic acid derivatives: a potential class of natural compounds for the management of lipid metabolism and obesity. *Nutr Metab.* (2016) 13:27. 10.1186/s12986-016-0080-3 27069498 PMC4827240

[79] Kumar N Goel N. Phenolic acids: natural versatile molecules with promising therapeutic applications. *Biotechnol Rep (Amst).* (2019) 24: e00370. 10.1016/j.btre.2019.e00370 PMC6734135 31516850

[80] Juurlink BH Azouz HJ Aldalati AM AlTinawi BMH Ganguly P. Hydroxybenzoic acid isomers and the cardiovascular system. *Nutr J.* (2014) 13:63. 10.1186/1475-2891-13-63 24943896 PMC4074389

[81] Shi Y Chen X Qiang S Su J Li J. Anti-oxidation and anti-inflammatory potency evaluation of ferulic acid derivatives obtained through virtual screening. *Int J Mol Sci*. (2021) 22:11305. 10.3390/ijms222111305 34768735 PMC8583578

[82] Yin ZN Wu WJ Sun CZ Liu HF Chen WB Zhan QP Antioxidant and anti-inflammatory capacity of ferulic acid released from wheat bran by solid-state fermentation of aspergillus niger. *Biomed Environ Sci.* (2019) 32:11–21. 10.3967/bes2019.002 30696535

[83] Vinayagam R Jayachandran M Xu B. Antidiabetic effects of simple phenolic acids: a comprehensive review. *Phytother Res.* (2016) 30:184–99. 10.1002/ptr.5528 26634804

[84] Salomone F Ivancovsky-Wajcman D Fliss-Isakov N Webb M Grosso G Godos J Higher phenolic acid intake independently associates with lower prevalence of insulin resistance and non-alcoholic fatty liver disease. *JHEP Rep.* (2020) 28:100069. 10.1016/j.jhepr.2020.100069 32195455 PMC7078532

[85] Rosa LS Silva NJA Soares NCP Monteiro MC Teodoro AJ. Anticancer properties of phenolic acids in colon cancer – a review. *J Nutr Food Sci.* (2016) 6:468. 10.4172/2155-9600.1000468

[86] Abotaleb M Liskova A Kubatka P Büsselberg D. Therapeutic potential of plant phenolic acids in the treatment of cancer. *Biomolecules.* (2020) 10:221. 10.3390/biom10020221 32028623 PMC7072661

[87] Altayli E Koru Ö Öngörü Ö Íde T Açikel C Sarper M An *in vitro* and *in vivo* investigation of the cytotoxic effects of caffeic acid (3,4-dihydroxycinnamic acid) phenethyl ester and bortezomib in multiple myeloma cells. *Turk J Med Sci.* (2015) 45:38–46. 10.3906/sag-1401-127 25790528

[88] Murugesan A Lassalle-Claux G Hogan L Vaillancourt E Selka A Luiker K Antimyeloma potential of caffeic acid phenethyl ester and its analogues through Sp1 mediated downregulation of IKZF1-IRF4-MYC Axis. *J Nat Prod.* (2020) 83:3526–35 10.1021/acs.jnatprod.0c00350 33210536

[89] Gu R Zhang M Meng H Xu D Xie Y. Gallic acid targets acute myeloid leukemia via Akt/mTOR-dependent mitochondrial respiration inhibition. *Biomed Pharmacother.* (2018) 105:491–7. 10.1016/j.biopha.2018.05.158 29883944

[90] Sourani Z Pourgheysari B Beshkar P Shirzad H Shirzad M. Gallic acid inhibits proliferation and induces apoptosis in lymphoblastic leukemia cell line (C121). *Iran J Med Sci.* (2016) 41:525–30. 27853333 PMC5106568

[91] Bellavia D Caradonna F Dico E Costa V Carina V De Luca A Non-flavonoid polyphenols in osteoporosis: preclinical evidence. *Trends Endocrinol Metab.* (2021) 32:515–29. 10.1016/j.tem.2021.03.008 33895073

[92] Akinwumi BC Bordun KM Anderson HD. Biological activities of stilbenoids. *Int J Mol Sci.* (2018) 19:792. 10.3390/ijms19030792 29522491 PMC5877653

[93] Yang T Fang L Sanders S Jayanthi S Rajan G Podicheti R Stilbenoid prenyltransferases define key steps in the diversification of peanut phytoalexins. *J Biol Chem.* (2018) 293:28–46. 10.1074/jbc.RA117.000564 29158266 PMC5766904

[94] El Khawand T Courtois A Valls J Richard T Krisa S. A review of dietary stilbenes: sources and bioavailability. *Phytochem Rev.* (2018) 17:1007–29. 10.1007/s11101-018-9578-9

[95] Seyed MA Jantan I Bukhari SN Vijayaraghavan KA. Comprehensive review on the chemotherapeutic potential of piceatannol for cancer treatment, with mechanistic insights. *J Agric Food Chem.* (2016) 64:725–37. 10.1021/acs.jafc.5b05993 26758628

[96] Eräsalo H Hämäläinen M Leppänen T Mäki-Opas I Laavola M Haavikko R Natural stilbenoids have anti-inflammatory properties *in vivo* and down-regulate the production of inflammatory mediators NO, IL6, and MCP1 possibly in a PI3K/Akt-dependent manner. *J Nat Prod.* (2018) 81:1131–42. 10.1021/acs.jnatprod.7b00384 29726680

[97] Mattio LM Catinella G Dallavalle S Pinto A. Stilbenoids: a natural arsenal against bacterial pathogens. *Antibiotics.* (2020) 9:336 10.3390/antibiotics9060336 32570824 PMC7345618

[98] Sirerol JA Rodríguez ML Mena S Asensi MA Estrela JM Ortega AL. Role of natural stilbenes in the prevention of cancer. *Oxid Med Cell Longev.* (2016) 2016:3128951. 10.1155/2016/3128951 26798416 PMC4698548

[99] Subedi L Teli MK Lee JH Gaire BP Kim MH Kim SYA. Stilbenoid isorhapontigenin as a potential anti-cancer agent against breast cancer through inhibiting sphingosine kinases/tubulin stabilization. *Cancers (Basel).* (2019) 11:1947. 10.3390/cancers11121947 31817453 PMC6966567

[100] Kirabo A Embury J Kiss R Polgár T Gali M Majumder A The stilbenoid tyrosine kinase inhibitor, G6, suppresses Jak2-V617F-mediated human pathological cell growth *in vitro* and *in vivo*. *J Biol Chem.* (2011) 286:4280–91. 10.1074/jbc.M110.200774 21127060 PMC3039371

[101] Lin H Cheng J Mu W Zhou J Zhu L. Advances in universal CAR-T cell therapy. *Front Immunol.* (2021) 12:744823. 10.3389/fimmu.2021.744823 34691052 PMC8526896

[102] Möhn N Bonda V Grote-Levi L Panagiota V Fröhlich T Schultze-Florey C Neurological management and work-up of neurotoxicity associated with CAR T cell therapy. *Neurol Res Pract.* (2022) 4:1. 10.1186/s42466-021-00166-5 35000613 PMC8744256

[103] Belin C Devic P Ayrignac X Dos Santos A Paix A Sirven-Villaros L Description of neurotoxicity in a series of patients treated with CAR T-cell therapy. *Sci Rep.* (2020) 10:18997. 10.1038/s41598-020-76055-9 33149178 PMC7642402

[104] Stock S Kluever AK Endres S Kobold S. Enhanced chimeric antigen receptor T cell therapy through co-application of synergistic combination partners. *Biomedicines.* (2022) 10:307. 10.3390/biomedicines10020307 35203517 PMC8869718

[105] Courtois G Gilmore TD. Mutations in the NF-kappaB signaling pathway: implications for human disease. *Oncogene.* (2006) 25:6831–43. 10.1038/sj.onc.1209939 17072331

[106] Vrábel D Pour L Ševčíková S. The impact of NF-κB signaling on pathogenesis and current treatment strategies in multiple myeloma. *Blood Rev.* (2019) 34:56–66. 10.1016/j.blre.2018.11.003 30501907

[107] Chauhan A Ul Islam A Prakash H Singh S. Phytochemicals targeting NF-κB signaling: potential anti-cancer interventions. *J Pharm Anal.* (2022) 12:394–405. 10.1016/j.jpha.2021.07.002 35811622 PMC9257438

[108] Takeda T Tsubaki M Kino T Yamagishi M Iida M Itoh T Mangiferin induces apoptosis in multiple myeloma cell lines by suppressing the activation of nuclear factor kappa B-inducing kinase. *Chem Biol Interact.* (2016) 251:26–33. 10.1016/j.cbi.2016.03.018 26996543

[109] Gomez-Pinillos A Ferrari AC. mTOR signaling pathway and mTOR inhibitors in cancer therapy. *Hematol Oncol Clin North Am.* (2012) 26:483–505. 10.1016/j.hoc.2012.02.014 22520976

[110] Hoxhaj G Manning BD. The PI3K-AKT network at the interface of oncogenic signalling and cancer metabolism. *Nat Rev Cancer.* (2020) 20:74–88. 10.1038/s41568-019-0216-7 31686003 PMC7314312

[111] Hawkins PT Stephens LR. PI3K signalling in inflammation. *Biochim Biophys Acta.* (2015) 1851:882–97. 10.1016/j.bbalip.2014.12.006 25514767

[112] Okkenhaug K Burger JA. PI3K signaling in normal B cells and chronic lymphocytic leukemia (CLL). *Curr Top Microbiol Immunol.* (2016) 393:123–42 26350103 10.1007/82_2015_484 PMC4704136

[113] Hus I Puła B Robak T. PI3K inhibitors for the treatment of chronic lymphocytic leukemia: current status and future perspectives. *Cancers.* (2022) 14:1571. 10.3390/cancers14061571 35326722 PMC8945984

[114] Kaneda MM Messer KS Ralainirina N Li H Leem CJ Gorjestani S PI3Kγ is a molecular switch that controls immune suppression. *Nature.* (2016) 539:437–42. 27642729 10.1038/nature19834 PMC5479689

[115] Ramadani F Bolland DJ Garcon F Emery JL Vanhaesebroeck B Corcoran AE The PI3K isoforms p110alpha and p110delta are essential for pre-B cell receptor signaling and B cell development. *Sci Signal.* (2010) 3:ra60. 10.1126/scisignal.2001104 20699475 PMC3540743

[116] Sarbassov DD Ali SM Sengupta S Sheen JH Hsu PP Bagley AF Prolonged rapamycin treatment inhibits mTORC2 assembly and Akt/PKB. *Molecular Cell.* (2006) 22:159–68. 10.1016/j.molcel.2006.03.029 16603397

[117] Jabbour E Ottmann OG Deininger M Hochhaus A. Targeting the phosphoinositide 3-kinase pathway in hematologic malignancies. *Haematologica.* (2014) 99:7–18. 10.3324/haematol.2013.087171 24425689 PMC4007928

[118] Mukohara T. PI3K mutations in breast cancer: prognostic and therapeutic implications. *Breast Cancer.* (2015) 7:111–23 26028978 10.2147/BCTT.S60696 PMC4440424

[119] Lamming DW. Inhibition of the mechanistic target of rapamycin (mTOR)-rapamycin and beyond. *Cold Spring Harb Perspect Med.* (2016) 6:a025924. 10.1101/cshperspect.a025924 27048303 PMC4852795

[120] Caron A Richard D Laplante M. The roles of mTOR complexes in lipid metabolism. *Annu Rev Nutr.* (2015) 35:321–48. 10.1146/annurev-nutr-071714-034355 26185979

[121] Kim YC Guan KL. mTOR: a pharmacologic target for autophagy regulation. *J Clin Invest.* (2015) 125:25–32. 10.1172/JCI73939 25654547 PMC4382265

[122] Martina JA Chen Y Gucek M Puertollano R. MTORC1 functions as a transcriptional regulator of autophagy by preventing nuclear transport of TFEB. *Autophagy.* (2012) 8:903–14 22576015 10.4161/auto.19653 PMC3427256

[123] Yuan T Yang Y Chen J Li W Li W Zhang Q Regulation of PI3K signaling in T-cell acute lymphoblastic leukemia: a novel PTEN/Ikaros/miR-26b mechanism reveals a critical targetable role for PIK3CD. *Leukemia.* (2017) 31:2355–64. 10.1038/leu.2017.80 28280276 PMC5986278

[124] Psyrri A Papageorgiou S Liakata E Scorilas A Rontogianni D Kontos CK Phosphatidylinositol 3′-kinase catalytic subunit alpha gene amplification contributes to the pathogenesis of mantle cell lymphoma. *Clin Cancer Res.* (2009) 15:5724–32. 10.1158/1078-0432.CCR-08-3215 19723646

[125] Yang J Nie J Ma X Wei Y Peng Y Wei X. Targeting PI3K in cancer: mechanisms and advances in clinical trials. *Mol Cancer.* (2019) 18:26. 10.1186/s12943-019-0954-x 30782187 PMC6379961

[126] Berning P Lenz G. The role of PI3K inhibitors in the treatment of malignant lymphomas. *Leuk Lymphoma.* (2021) 62:517–27. 10.1080/10428194.2020.1839654 33135516

[127] Martini M De Santis MC Braccini L Gulluni F Hirsch E. PI3K/AKT signaling pathway and cancer: an updated review. *Ann Med.* (2014) 46:372–83 24897931 10.3109/07853890.2014.912836

[128] Pons-Tostivint E Thibault B Guillermet-Guibert J. Targeting PI3K signaling in combination cancer therapy. *Trends Cancer.* (2017) 3:454–69. 10.1016/j.trecan.2017.04.002 28718419

[129] Zhang J Han X Hu X Jin F Gao Z Yin L IDO1 impairs NK cell cytotoxicity by decreasing NKG2D/NKG2DLs via promoting miR-18a. *Mol Immunol.* (2018) 103:144–55. 10.1016/j.molimm.2018.09.011 30268986

[130] Zou Z Tao T Li H Zhu X. mTOR signaling pathway and mTOR inhibitors in cancer: progress and challenges. *Cell Biosci.* (2020) 10:31. 10.1186/s13578-020-00396-1 32175074 PMC7063815

[131] Coleman N Moyers JT Harbery A Vivanco I Yap TA. Clinical development of AKT inhibitors and associated predictive biomarkers to guide patient treatment in cancer medicine. *Pharmgenomics Pers Med.* (2021) 14:1517–35. 10.2147/PGPM.S305068 34858045 PMC8630372

[132] Maurya AK Vinayak M. Quercetin attenuates cell survival, inflammation, and angiogenesis via modulation of AKT signaling in murine T-cell lymphoma. *Nutr Cancer.* (2017) 69:470–80. 10.1080/01635581.2017.1267775 28107044

[133] Hideshima T Catley L Raje N Chauhan D Podar K Mitsiades C Inhibition of Akt induces significant downregulation of surviving and cytotoxicity in human multiple myeloma cells. *Brit J Hematol.* (2007) 138:783–91. 10.1111/j.1365-2141.2007.06714.x 17760810

[134] Kharas MG Okabe R Ganis JJ Gozo M Khandan T Paktinat M Constitutively active AKT depletes hematopoietic stem cells and induces leukemia in mice. *Blood.* (2010) 115:1406–15. 10.1182/blood-2009-06-229443 20008787 PMC2826762

[135] Kuttikrishnan S Siveen KS Prabhu KS Khan AQ Ahmed EI Akhtar S Curcumin induces apoptotic cell death via inhibition of PI3-Kinase/AKT pathway in B-precursor acute lymphoblastic leukemia. *Front Oncol.* (2019) 9:484. 10.3389/fonc.2019.00484 31275848 PMC6593070

[136] Ghosh AK Kay NE Secreto CR Shanafelt TD. Curcumin inhibits prosurvival pathways in chronic lymphocytic leukemia B cells and may overcome their stromal protection in combination with EGCG. *Clin Cancer Res.* (2009) 15:1250–58. 10.1158/1078-0432.CCR-08-1511 19228728 PMC3893060

[137] Chang NC. Autophagy and stem cells: self-eating for self-renewal. *Front Cell Dev Biol.* (2020) 4:138. 10.3389/fcell.2020.00138 32195258 PMC7065261

[138] Yu X Long YC Shen HM. Differential regulatory functions of three classes of phosphatidylinositol and phosphoinositide 3-kinases in autophagy. *Autophagy.* (2015) 11:1711–28. 10.1080/15548627.2015.1043076 26018563 PMC4824607

[139] Paquette M El-Houjeiri L Pause A. mTOR Pathways in cancer and autophagy. *Cancers.* (2018) 10:18 10.3390/cancers10010018 PMC5789368 29329237

[140] Siedlecka-Kroplewska K Wozniak M Kmiec Z. The wine polyphenol resveratrol modulates autophagy and induces apoptosis in MOLT-4 and HL-60 human leukemia cells. *J Physiol Pharmacol.* (2019) 70:825–38. 10.26402/jpp.2019.6.02 32084644

[141] Puissant A Robert G Fenouille N Luciano F Cassuto JP Raynaud S Resveratrol promotes autophagic cell death in chronic myelogenous leukemia cells via JNK-mediated p62/SQSTM1 expression and AMPK activation. *Cancer Res.* (2010) 70:1042–52. 10.1158/0008-5472.CAN-09-3537 20103647

[142] Ali Azzwali AAA Azab AE Alfourti AMAB. Induction of autophagy in human myeloid and lymphoid leukaemia cell line by using polyphenols alone and combined with a stander chemotherapy. *EASJ Pharm Pharmacol.* (2019) 1:64–70. 10.36349/easjpp.2019.v01i03.001

[143] Guo Y Shan QQ Gong PY Wang SC. The autophagy induced by curcumin via MEK/ERK pathway plays an early anti-leukemia role in human Philadelphia chromosome-positive acute lymphoblastic leukemia SUP-B15 cells. *J Cancer Res Ther.* (2018) 14:S125–31. 10.4103/0973-1482.172111 29578162

[144] Ebi H Costa C Faber AC Nishtala M Kotani H Juric D PI3K regulates MEK/ERK signaling in breast cancer via the Rac-GEF, P-Rex1. *Proc Natl Acad Sci USA.* (2013) 110:21124–29. 10.1073/pnas.1314124110 24327733 PMC3876254

[145] Winter JN Jefferson LS Kimball SR. ERK and Akt signaling pathways function through parallel mechanisms to promote mTORC1 signaling. *Am J Physiol Cell Physiol.* (2011) 300:C1172–80. 10.1152/ajpcell.00504.2010 21289294 PMC3093949

[146] Jia YL Li J Qin ZH Liang ZQ. Autophagic and apoptotic mechanisms of curcumin-induced death in K562 cells. *J Asian Nat Prod Res.* (2009) 11:918–28. 10.1080/10286020903264077 20183254

[147] Gu HF Li HZ Tang YL Tang XQ Zheng XL Liao DF. Nicotinate-curcumin impedes foam cell formation from THP-1 cells through restoring autophagy flux. *PLoS One.* (2016) 11:e0154820. 10.1371/journal.pone.0154820 27128486 PMC4851383

[148] Zaman S Wang R Gandhi V. Targeting the apoptosis pathway in hematologic malignancies. *Leuk Lymphoma.* (2014) 55:1980–92. 10.3109/10428194.2013.855307 24295132 PMC4152229

[149] Kaufmann SH Karp JE Svingen PA Krajewski S Burke PJ Gore SD Elevated expression of the apoptotic regulator Mcl-1 at the time of leukemic relapse. *Blood.* (1998) 91:991–1000. 9446661

[150] Wu H Medeiros LJ Young KH. Apoptosis signaling and BCL-2 pathways provide opportunities for novel targeted therapeutic strategies in hematologic malignances. *Blood Rev.* (2018) 32:8–28. 10.1016/j.blre.2017.08.004 28802908

[151] Schleich K Krammer PH Lavrik IN. The chains of death: a new view on caspase-8 activation at the DISC. *Cell Cycle.* (2013) 12:193–4. 10.4161/cc.23464 23287476 PMC3575441

[152] LaCasse EC Mahoney DJ Cheung HH Plenchette S Baird S Korneluk RG. IAP-targeted therapies for cancer. *Oncogene.* (2008) 27:6252–75. 10.1038/onc.2008.302 18931692

[153] Obexer P Ausserlechner MJ. X-linked inhibitor of apoptosis protein – a critical death resistance regulator and therapeutic target for personalized cancer therapy. *Front Oncol.* (2014) 4:197. 10.3389/fonc.2014.00197 25120954 PMC4112792

[154] Jiang H Fan D Zhou G Li X Deng H. Phosphatidylinositol 3-kinase inhibitor(LY294002) induces apoptosis of human nasopharyngeal carcinoma *in vitro* and *in vivo*. *J Exp Clin Cancer Res*. (2010) 29:34. 10.1186/1756-9966-29-34 20412566 PMC2873422

[155] Ahmad A Biersack B Li Y Kong D Bao B Schobert R Targeted regulation of PI3K/Akt/mTOR/NF-κB signaling by indole compounds and their derivatives: mechanistic details and biological implications for cancer therapy. *Anticancer Agents Med Chem.* (2013) 13:1002–13. 10.2174/18715206113139990078 23272910 PMC3901097

[156] Pugazhenthi S Nesterova A Sable C Heidenreich KA Boxer LM Heasley LE Akt/protein kinase B up-regulates Bcl-2 expression through cAMP-response element-binding protein. *J Biol Chem.* (2000) 275:10761–6. 10.1074/jbc.275.15.10761 10753867

[157] Dai Y Jin S Li X Wang D. The involvement of Bcl-2 family proteins in AKT-regulated cell survival in cisplatin resistant epithelial ovarian cancer. *Oncotarget.* (2017) 8:1354–68. 10.18632/oncotarget.13817 27935869 PMC5352061

[158] Kizilboga T Baskale EA Yildiz J Akcay IM Zemheri E Can ND. Bag-1 stimulates Bad phosphorylation through activation of Akt and Raf kinases to mediate cell survival in breast cancer. *BMC Cancer.* (2019) 19:1254. 10.1186/s12885-019-6477-4 31883527 PMC6935482

[159] Nitulescu GM Van De Venter M Nitulescu G Ungurianu A Juzenas P. The Akt pathway in oncology therapy and beyond (Review). *Int J Oncol.* (2018) 53:2319–31 30334567 10.3892/ijo.2018.4597 PMC6203150

[160] Boucher MJ Morisset J Vachon PH Reed JC Lainé J Rivard N. MEK/ERK signaling pathway regulates the expression of Bcl-2, Bcl-X(L), and Mcl-1 and promotes survival of human pancreatic cancer cells. *J Cell Biochem.* (2000) 79:355–69. 10972974

[161] Cook SJ Stuart K Gilley R Sale MJ. Control of cell death and mitochondrial fission by ERK1/2 MAP kinase signalling. *FEBS J.* (2017) 284:4177–95. 10.1111/febs.14122 28548464 PMC6193418

[162] O’Reilly LA Kruse EA Puthalakath H Kelly PN Kaufmann T Huang DC MEK/ERK-mediated phosphorylation of Bim is required to ensure survival of T and B lymphocytes during mitogenic stimulation. *J Immunol.* (2009) 183:261–9 10.4049/jimmunol.0803853 19542438 PMC2950174

[163] Billard C. Apoptosis inducers in chronic lymphocytic leukemia. *Oncotarget.* (2014) 5:309–25. 10.18632/oncotarget.1480 24525395 PMC3964209

[164] Pellecchia M Reed JC. Inhibition of anti-apoptotic Bcl-2 family proteins by natural polyphenols: new avenues for cancer chemoprevention and chemotherapy. *Curr Pharm Des.* (2004) 10:1387–98. 10.2174/1381612043384880 15134489

[165] Reed JC Pellecchia M. Apoptosis-based therapies for hematologic malignancies. *Blood.* (2005) 106:408–18. 10.1182/blood-2004-07-2761 15797997

[166] Ni Z Dai X Wang B Ding W Cheng P Xu L Lian J He F Natural Bcl-2 inhibitor (–)- gossypol induces protective autophagy via reactive oxygen species-high mobility group box 1 pathway in Burkitt lymphoma. *Leuk Lymphoma.* (2013) 54:2263–8 10.3109/10428194.2013.775437 23398207

[167] Balakrishnan K Burger JA Wierda WG Gandhi V. AT-101 induces apoptosis in CLL B cells and overcomes stromal cell-mediated Mcl-1 induction and drug resistance. *Blood.* (2009) 113:149–53. 10.1182/blood-2008-02-138560 18836097 PMC2614629

[168] Valentin R Grabow S Davids MS. The rise of apoptosis: targeting apoptosis in hematologic malignancies. *Blood.* (2018) 132:1248–64. 10.1182/blood-2018-02-791350 30012635

[169] Liu WH Chang LS. Piceatannol induces Fas and FasL up-regulation in human leukemia U937 cells via Ca2+/p38alpha MAPK-mediated activation of c-Jun and ATF-2 pathways. *Int J Biochem Cell Biol.* (2010) 42:1498–506. 10.1016/j.biocel.2010.05.007 20580678

[170] Li Q Yue Y Chen L Xu C Wang Y Du L Resveratrol sensitizes carfilzomib-induced apoptosis via promoting oxidative stress in multiple myeloma cells. *Front Pharmacol.* (2018) 9:334. 10.3389/fphar.2018.00334 29867453 PMC5961230

[171] Wu XP Xiong M Xu CS Duan LN Dong YQ Luo Y Resveratrol induces apoptosis of human chronic myelogenous leukemia cells *in vitro* through p38 and JNK-regulated H2AX phosphorylation. *Acta Pharmacol Sin.* (2015) 36:353–61. 10.1038/aps.2014.132 25619392 PMC4349923

[172] Chen R Zhang H Liu P Wu X Chen B. Gambogenic acid synergistically potentiates bortezomib-induced apoptosis in multiple myeloma. *J Cancer.* (2017) 8:839–51. 10.7150/jca.17657 28382147 PMC5381173

[173] Allegra A Speciale A Molonia MS Guglielmo L Musolino C Ferlazzo G Curcumin ameliorates the *in vitro* efficacy of carfilzomib in human multiple myeloma U266 cells targeting p53 and NF-κB pathways. *Toxicol In Vitro.* (2018) 47:186–94. 10.1016/j.tiv.2017.12.001 29223572

[174] Ramakrishna R Diamond TH Alexander W Manoharan A Golombick T. Use of curcumin in multiple myeloma patients intolerant of steroid therapy. *Clin Case Rep.* (2020) 8:739–44. 10.1002/ccr3.2735 32274049 PMC7141712

[175] Zaidi A Lai M Cavenagh J. Long-term stabilisation of myeloma with curcumin. *BMJ Case Rep.* (2017) 2017:bcr2016218148. 10.1136/bcr-2016-218148 28413157 PMC5753731

[176] Han MH Lee WS Nagappan A Kim HJ Park C Kim GY Polyphenols from Korean prostrate spurge Euphorbia supina induce apoptosis through the Fas-associated extrinsic pathway and activation of ERK in human leukemic U937 cells. *Oncol Rep.* (2016) 36:99–107. 10.3892/or.2016.4778 27122127 PMC4899010

[177] Visentin A Frezzato F Severin F Imbergamo S Pravato S Romano Gargarella L Lights and shade of next-generation PI3K inhibitors in chronic lymphocytic leukemia. *Onco Targets Ther.* (2020) 13:9679–88. 10.2147/OTT.S268899 33061448 PMC7532889

[178] Schafer KA. The cell cycle: a review. *Vet Pathol.* (1998) 35:461–78. 10.1177/030098589803500601 9823588

[179] Stark GR Taylor WR. Analyzing the G2/M checkpoint. *Methods Mol Biol.* (2004) 280:51–82. 10.1385/1-59259-788-2:051 15187249

[180] Casimiro MC Crosariol M Loro E Li Z Pestell RG. Cyclins and cell cycle control in cancer and disease. *Genes Cancer.* (2012) 3:649–57. 10.1177/1947601913479022 23634253 PMC3636749

[181] Balakrishnan A Vyas A Deshpande K Vyas D. Pharmacological cyclin dependent kinase inhibitors: implications for colorectal cancer. *World J Gastroenterol.* (2016) 22:2159–64. 10.3748/wjg.v22.i7.2159 26900281 PMC4734993

[182] Aprelikova O Xiong Y Liu ET. Both p16 and p21 families of cyclin-dependent kinase (CDK) inhibitors block the phosphorylation of cyclin-dependent kinases by the CDK-activating kinase. *J Biol Chem.* (1995) 270:18195–7. 10.1074/jbc.270.31.18195 7629134

[183] Quereda V Porlan E Cañamero M Dubus P Malumbres M. An essential role for Ink4 and Cip/Kip cell-cycle inhibitors in preventing replicative stress. *Cell Death Differ.* (2016) 23:430–41. 10.1038/cdd.2015.112 26292757 PMC5072439

[184] Cerqueira A tín A Symonds CE Odajima J Dubus P Barbacid M Genetic characterization of the role of the Cip/Kip family of proteins as cyclin-dependent kinase inhibitors and assembly factors. *Mol Cell Biol.* (2014) 34:1452–9. 10.1128/MCB.01163-13 24515438 PMC3993583

[185] Lee EY Muller WJ. Oncogenes and tumor suppressor genes. *Cold Spring Harb Perspect Biol.* (2010) 2:a003236. 10.1101/cshperspect.a003236 20719876 PMC2944361

[186] Molica M Mazzone C Niscola P de Fabritiis P. TP53 mutations in acute myeloid leukemia: still a daunting challenge? *Front Oncol.* (2021) 10:610820. 10.3389/fonc.2020.610820 33628731 PMC7897660

[187] Orsmark-Pietras C Landberg N Lorenz F Uggla B Höglund M Lehmann S Clinical and genomic characterization of patients diagnosed with the provisional entity acute myeloid leukemia with BCR-ABL1, a Swedish population-based study. *Genes Chromosomes Cancer.* (2021) 60:426–33 10.1002/gcc.22936 33433047

[188] Henley SA Dick FA. The retinoblastoma family of proteins and their regulatory functions in the mammalian cell division cycle. *Cell Div.* (2012) 7:10. 10.1186/1747-1028-7-10 22417103 PMC3325851

[189] Chen J. The cell-cycle arrest and apoptotic functions of p53 in tumor initiation and progression. *Cold Spring Harb Perspect Med.* (2016) 6:a026104. 10.1101/cshperspect.a026104 26931810 PMC4772082

[190] Feroz W Sheikh AMA. Exploring the multiple roles of guardian of the genome: P53. *Egypt J Med Hum Genet.* (2020) 21:49. 10.1186/s43042-020-00089-x

[191] Sanford JD Yang J Han J Tollini LA Jin A Zhang Y. MDMX is essential for the regulation of p53 protein levels in the absence of a functional MDM2 C-terminal tail. *BMC Mol Cell Biol.* (2021) 22:46. 10.1186/s12860-021-00385-3 34551723 PMC8459461

[192] Gnanapradeepan K Basu S Barnoud T Budina-Kolomets A Kung CP Murphy ME. The p53 tumor suppressor in the control of metabolism and ferroptosis. *Front Endocrinol.* (2018) 9:124. 10.3389/fendo.2018.00124 29695998 PMC5904197

[193] Marei HE Althani A Afifi N Hasan A Caceci T Pozzoli G p53 signaling in cancer progression and therapy. *Cancer Cell Int.* (2021) 21:703 10.1186/s12935-021-02396-8 PMC8709944 34952583

[194] Rivlin N Brosh R Oren M Rotter V. Mutations in the p53 tumor suppressor gene: important milestones at the various steps of tumorigenesis. *Genes Cancer.* (2011) 2:466–74. 10.1177/1947601911408889 21779514 PMC3135636

[195] Peller S Rotter V. TP53 in hematological cancer: low incidence of mutations with significant clinical relevance. *Hum Mutat.* (2003) 21:277–84 12619113 10.1002/humu.10190

[196] Alkhatabi H Yasin EB Mirza Z Alserihi R Felimban R Elaimi A. TP53 Expression and Mutational Analysis in Hematological Malignancy in Jeddah, Saudi Arabia. *Diagnostics.* (2022) 12:724. 10.3390/diagnostics12030724 35328276 PMC8946951

[197] Canavese M Santo L Raje N. Cyclin dependent kinases in cancer: potential for therapeutic intervention. *Cancer Biol Ther.* (2012) 13:451–7. 10.4161/cbt.19589 22361734

[198] Yano S Miwa S Mii S Hiroshima Y Uehara F Yamamoto M Invading cancer cells are predominantly in G0/G1 resulting in chemoresistance demonstrated by real-time FUCCI imaging. *Cell Cycle.* (2014) 13:953–60. 10.4161/cc.27818 24552821 PMC3984318

[199] Pang W Li Y Guo W Shen H. Cyclin E: a potential treatment target to reverse cancer chemoresistance by regulating the cell cycle. *Am J Transl Res.* (2020) 12:5170–87 33042412 PMC7540110

[200] Mohanty S Tran T Sandoval N Mohanty A Bedell V Wu J Cyclin D1 promotes survival and chemoresistance by maintaining ATR and CHEK1 signaling in TP53-deficient mantle cell lymphoma cell lines. *Blood.* (2014) 124:5197. 10.1182/blood.V124.21.5197.5197

[201] Busa D Loja T Jeziskova I Folta A Mayer J Culen M. Palbociclib and ponatinib suppress acute myeloid leukemia in patient-derived xenograft. *Blood.* (2021) 138(Suppl. 1):4461. 10.1182/blood-2021-145972

[202] Uras IZ Walter GJ Scheicher R Bellutti F Prchal-Murphy M Tigan AS Palbociclib treatment of FLT3-ITD+ AML cells uncovers a kinase-dependent transcriptional regulation of FLT3 and PIM1 by CDK6. *Blood.* (2016) 127:2890–902. 10.1182/blood-2015-11-683581 27099147 PMC4920675

[203] Fröhling S Agrawal M Jahn N Fransecky LR Baldus CD Wäsch R CDK4/6 Inhibitor palbociclib for treatment of KMT2A-rearranged acute myeloid leukemia: interim analysis of the AMLSG 23-14 Trial. *Blood.* (2016) 128:1608. 10.1182/blood.V128.22.1608.1608

[204] Granato M Gilardini Montani MS Santarelli R D’Orazi G Faggioni A Cirone M. Apigenin, by activating p53 and inhibiting STAT3, modulates the balance between pro-apoptotic and pro-survival pathways to induce PEL cell death. *J Exp Clin Cancer Res.* (2017) 36:167. 10.1186/s13046-017-0632-z 29179721 PMC5704516

[205] Izuegbuna O Otunola GA Bradley G. GC-MS profiling and antineoplastic activity of pelargonium inquinans ait leaves on acute leukaemia cell lines U937 and Jurkat. *Nutr Cancer.* (2022) 74:1849–71. 10.1080/01635581.2021.1969417 34477039

[206] Shih YZ Huang AJ Hou CY Jiang CM Wu MC. The stimulating effects of polyphenol and protein fractions from jelly fig (Ficus awkeotsang Makino) achenes against proliferation of leukemia cells. *J Food Drug Anal.* (2017) 25:854–61. 10.1016/j.jfda.2016.10.015 28987362 PMC9328882

[207] Abubakar MB Abdullah WZ Sulaiman SA Ang BS. Polyphenols as key players for the antileukaemic effects of propolis. *Evid Based Complement Alternat Med.* (2014) 2014:371730. 10.1155/2014/371730 24772179 PMC3977507

[208] Choi CY Lim SC Lee TB Han SI. Molecular basis of resveratrol-induced resensitization of acquired drug-resistant cancer cells. *Nutrients.* (2022) 14:699. 10.3390/nu14030699 35277058 PMC8838003

[209] Syed DN Chamcheu JC Adhami VM Mukhtar H. Pomegranate extracts and cancer prevention: molecular and cellular activities. *Anticancer Agents Med Chem.* (2013) 13:1149–61. 10.2174/1871520611313080003 23094914 PMC4052369

[210] Dahlawi H Jordan-Mahy N Clench MR Le Maitre CL. Bioactive actions of pomegranate fruit extracts on leukemia cell lines *in vitro* hold promise for new therapeutic agents for leukemia. *Nutr Cancer.* (2012) 64:100–10. 10.1080/01635581.2012.630155 22098126

[211] Ceesay MM Vadher B Tinwell B Goderya R Sawicka E. Spontaneous remission of T lymphoblastic lymphoma. *J Clin Pathol.* (2008) 61:955–7. 10.1136/jcp.2008.056697 18663057

[212] Martínez-Castillo M Villegas-Sepúlveda N Meraz-Rios MA Hernández-Zavala A Berumen J Coleman MA Curcumin differentially affects cell cycle and cell death in acute and chronic myeloid leukemia cells. *Oncol Lett.* (2018) 15:6777–83 10.3892/ol.2018.8112 29616136 PMC5876431

[213] Zhou H Ning Y Zeng G Zhou C Ding X. Curcumin promotes cell cycle arrest and apoptosis of acute myeloid leukemia cells by inactivating AKT. *Oncol Rep.* (2021) 45:11. 10.3892/or.2021.7962 33649826 PMC7877002

[214] Devassy JG Nwachukwu ID Jones PJH. Curcumin and cancer: barriers to obtaining a health claim. *Nutr Rev.* (2015) 73:155–65. 10.1093/nutrit/nuu064 26024538

[215] Mahbub AA Le Maitre CL Haywood-Small SL Cross NA Jordan-Mahy N. Polyphenols act synergistically with doxorubicin and etoposide in leukaemia cell lines. *Cell Death Discov.* (2015) 1:15043 10.1038/cddiscovery.2015.43 27551472 PMC4979421

[216] Mahbub A Le Maitre C Haywood-Small S Cross N Jordan-Mahy N. Dietary polyphenols influence antimetabolite agents: methotrexate, 6-mercaptopurine and 5-fluorouracil in leukemia cell lines. *Oncotarget.* (2017) 8:104877–93 10.18632/oncotarget.20501 29285220 PMC5739607

[217] Ishikawa C Senba M Mori N. Butein inhibits NF-κB, AP-1 and Akt activation in adult T-cell leukemia/lymphoma. *Int J Oncol.* (2017) 51:633–43. 10.3892/ijo.2017.4026 28586006

[218] Savitz J. The kynurenine pathway: a finger in every pie. *Mol Psychiatry.* (2020) 25:131–47. 30980044 10.1038/s41380-019-0414-4 PMC6790159

[219] Davis I Liu A. What is the tryptophan kynurenine pathway and why is it important to neurotherapeutics?. *Expert Rev Neurother.* (2015) 15:719–21. 10.1586/14737175.2015.1049999 26004930 PMC4482796

[220] Zulfiqar B Mahroo A Nasir K Farooq RK Jalal N Rashid MU Nanomedicine and cancer immunotherapy: focus on indoleamine 2,3-dioxygenase inhibitors. *Onco Targets Ther.* (2017) 10:463–76. 10.2147/OTT.S119362 28176942 PMC5268369

[221] Vogel CFA Wu D Goth SR Baek J Lollies A Domhardt R Aryl hydrocarbon receptor signaling regulates NF-κB RelB activation during dendritic-cell differentiation. *Immunol Cell Biol.* (2013) 91:568–75. 10.1038/icb.2013.43 23999131 PMC3806313

[222] Pallotta MT Fallarino F Matino D Macchiarulo A Orabona C. AhR-mediated, non-genomic modulation of IDO1 function. *Front Immunol.* (2014) 5:497. 10.3389/fimmu.2014.00497 25360135 PMC4197771

[223] Dixon JR Jung I Selvaraj S Shen Y Antosiewicz-Bourget JE Lee AY Chromatin architecture reorganization during stem cell differentiation. *Nature.* (2015) 518:331–6. 10.1038/nature14222 25693564 PMC4515363

[224] Hucke C MacKenzie CR Adjogble KD Takikawa O Däubener W. Nitric oxide-mediated regulation of gamma interferon-induced bacteriostasis: inhibition and degradation of human indoleamine 2,3-dioxygenase. *Infect Immun.* (2004) 72:2723–30. 10.1128/IAI.72.5.2723-2730.2004 15102781 PMC387869

[225] Banzola I Mengus C Wyler S Hudolin T Manzella G Chiarugi AETAL. Expression of indoleamine 2,3-dioxygenase induced by IFN-γ and TNF-α as potential biomarker of prostate cancer progression. *Front Immunol.* (2018) 9:1051. 10.3389/fimmu.2018.01051 29896191 PMC5986916

[226] Sinclair LV Neyens D Ramsay G Taylor PM Cantrell DA. Single cell analysis of kynurenine and System L amino acid transport in T cells. *Nat Commun.* (2018) 9:1981. 10.1038/s41467-018-04366-7 29773791 PMC5958064

[227] Mezrich JD Fechner JH Zhang X Johnson BP Burlingham WJ Bradfield CA An interaction between kynurenine and the aryl hydrocarbon receptor can generate regulatory T cells. *J Immunol.* (2010) 185:3190–8 20720200 10.4049/jimmunol.0903670 PMC2952546

[228] Zaher SS Germain C Fu H Larkin DF George AJ. 3-hydroxykynurenine suppresses CD4+ T-cell proliferation, induces T-regulatory-cell development, and prolongs corneal allograft survival. *Invest Ophthalmol Vis Sci.* (2011) 52:2640–8. 10.1167/iovs.10-5793 21212175 PMC3088555

[229] Hayashi T Mo JH Gong X Rossetto C Jang A Beck L 3-Hydroxyanthranilic acid inhibits PDK1 activation and suppresses experimental asthma by inducing T cell apoptosis. *Proc Natl Acad Sci USA.* (2007) 104:18619–24. 10.1073/pnas.0709261104 18003900 PMC2141826

[230] Lee K Kwak JH Pyo S. Inhibition of LPS-induced inflammatory mediators by 3-hydroxyanthranilic acid in macrophages through suppression of PI3K/NF-κB signaling pathways. *Food Funct.* (2016) 7:3073–82. 10.1039/c6fo00187d 27264984

[231] Sahm F Oezen I Opitz AC Radlwimmer B von Deimling A Ahrendt T The endogenous tryptophan metabolite and nad^+^ precursor quinolinic acid confers resistance of gliomas to oxidative stress. *Cancer Res.* (2013) 73:3225–34. 10.1158/0008-5472.CAN-12-3831 23548271

[232] Prodinger J Loacker LJ Schmidt RL Ratzinger F Greiner G Witzeneder N The tryptophan metabolite picolinic acid suppresses proliferation and metabolic activity of CD4+ T cells and inhibits c-Myc activation. *J Leukoc Biol.* (2016) 99:583–94. 10.1189/jlb.3A0315-135R 26497245

[233] Yoshio S Sugiyama M Shoji H Mano Y Mita E Okamoto T Indoleamine-2,3-dioxygenase as an effector and an indicator of protective immune responses in patients with acute hepatitis B. *Hepatology.* (2016) 63:83–94. 10.1002/hep.28282 26458241

[234] Dos Santos RO da Cruz MGS Lopes SCP Oliveira LB Nogueira PA Lima ES A First *Plasmodium vivax* Natural Infection Induces Increased Activity of the Interferon Gamma-Driven Tryptophan Catabolism Pathway. *Front Microbiol.* (2020) 11:400. 10.3389/fmicb.2020.00400 32256470 PMC7089964

[235] Savitz J. The kynurenine pathway: a finger in every pie. *Mol Psychiatry.* (2020) 25:131–47. 10.1038/s41380-019-0414-4 30980044 PMC6790159

[236] Zhang L Ovchinnikova O Jönsson A Lundberg AM Berg M Hansson GK The tryptophan metabolite 3-hydroxyanthranilic acid lowers plasma lipids and decreases atherosclerosis in hypercholesterolaemic mice. *Eur Heart J.* (2012) 33:2025–34. 10.1093/eurheartj/ehs175 22711758

[237] Hornyák L Dobos N Koncz G Karányi Z Páll D Szabó Z The role of indoleamine-2,3-dioxygenase in cancer development, diagnostics, and therapy. *Front Immunol.* (2018) 9:151. 10.3389/fimmu.2018.00151 29445380 PMC5797779

[238] Prendergast GC Smith C Thomas S Mandik-Nayak L Laury-Kleintop L Metz R Indoleamine 2,3-dioxygenase pathways of pathogenic inflammation and immune escape in cancer. *Cancer Immunol Immunother.* (2014) 63:721–35. 10.1007/s00262-014-1549-4 24711084 PMC4384696

[239] Löb S Königsrainer A Zieker D Brücher BLDM Rammensee HG Opelz G IDO1 and IDO2 are expressed in human tumors: levo- but not dextro-1-methyl tryptophan inhibits tryptophan catabolism. *Cancer Immunol Immunother.* (2009) 58:153–7 10.1007/s00262-008-0513-6 18418598 PMC11030193

[240] Fang X Guo L Xing Z Shi L Liang H Li A IDO1 can impair NK cells function against non-small cell lung cancer by downregulation of NKG2D Ligand via ADAM10. *Pharmacol Res.* (2022) 177:106132. 10.1016/j.phrs.2022.106132 35183714

[241] Salminen A. Role of indoleamine 2,3-dioxygenase 1 (IDO1) and kynurenine pathway in the regulation of the aging process. *Ageing Res Rev.* (2022) 75:101573. 10.1016/j.arr.2022.101573 35085834

[242] Holmgaard RB Zain D Li Y Gasmi B Munn DH Allison JP Tumor-expressed IDO recruits and activates MDSCs in a treg-dependent manner. *Cell Rep.* (2015) 13:412–24. 10.1016/j.celrep.2015.08.077 26411680 PMC5013825

[243] Curti A Aluigi M Pandolfi S Ferri E Isidori A Salvestrini V Acute myeloid leukemia cells constitutively express the immunoregulatory enzyme indoleamine 2,3-dioxygenase. *Leukemia.* (2007) 21:353–5. 10.1038/sj.leu.2404485 17170728

[244] Wells G Kennedy PT Dahal LN. Investigating the role of indoleamine 2,3-dioxygenase in acute myeloid leukemia: a systematic review. *Front Immunol.* (2021) 12:651687. 10.3389/fimmu.2021.651687 33777052 PMC7988196

[245] Ragaini S Wagner S Marconi G Parisi S Sartor C Nanni J. An IDO1-related immune gene signature predicts overall survival in acute myeloid leukemia. *Blood Adv.* (2022) 6:87–99. 10.1182/bloodadvances.2021004878 34535017 PMC8753212

[246] Rutella S Folgiero V Filippini P Bertaina V Masetti R Zecca M. Indoleamine 2,3-dioxygenase-1 (IDO1) expression by childhood acute myeloid leukemias inhibits T-cell production of IFN-γ and confers an unfavorable prognosis. *J Immunother Cancer.* (2013) 1(Suppl. 1):172. 10.1186/2051-1426-1-S1-P172

[247] Folgiero V Goffredo BM Filippini P Masetti R Bonanno G Caruso R Indoleamine 2,3-dioxygenase 1 (IDO1) activity in leukemia blasts correlates with poor outcome in childhood acute myeloid leukemia. *Oncotarget.* (2014) 5:2052–64. 10.18632/oncotarget.1504 24903009 PMC4039144

[248] Atene CG Fiorcari S Mesini N Alboni S tinelli S Maccaferri M Indoleamine 2, 3-dioxygenase 1 mediates survival signals in chronic lymphocytic leukemia *via* kynurenine/aryl hydrocarbon receptor-mediated mcl1 modulation. *Front Immunol.* (2022) 13:832263. 10.3389/fimmu.2022.832263 35371054 PMC8971515

[249] Robinson CM Hale PT Carlin JM. The role of IFN-gamma and TNF-alpha-responsive regulatory elements in the synergistic induction of indoleamine dioxygenase. *J Interferon Cytokine Res.* (2005) 25:20–30. 10.1089/jir.2005.25.20 15684619 PMC1488823

[250] Jung ID Lee MG Chang JH Lee JS Jeong YI Lee CM. Blockade of indoleamine 2,3-dioxygenase protects mice against lipopolysaccharide-induced endotoxin shock. *J Immunol.* (2009) 182:3146–54. 10.4049/jimmunol.0803104 19234212

[251] Lee SY Choi HK Lee KJ Jung JY Hur GY Jung KH The immune tolerance of cancer is mediated by IDO that is inhibited by COX-2 inhibitors through regulatory T cells. *J Immunother.* (2009) 32:22–8. 10.1097/CJI.0b013e31818ac2f7 19307990

[252] Iachininoto MG Nuzzolo ER Di Maggio A Bonanno G Mariotti A Procoli A. COX-2 inhibition suppresses the interferon-γ-induced expression of indoleamine 2,3-dioxygenase (IDO) in human leukemia cell lines. *Blood.* (2008) 112:1623. 10.1182/blood.V112.11.1623.1623

[253] Schroecksnadel K Winkler C Wirleitner B Schennach H Fuchs D. Aspirin down-regulates tryptophan degradation in stimulated human peripheral blood mononuclear cells *in vitro*. *Clin Exp Immunol.* (2005) 140:41–5. 10.1111/j.1365-2249.2005.02746.x 15762873 PMC1809338

[254] Gostner JM Schroecksnadel S Jenny M Klein A Ueberall F Schennach H Coffee extracts suppress tryptophan breakdown in mitogen-stimulated peripheral blood mononuclear cells. *J Am Coll Nutr.* (2015) 34:212–23. 10.1080/07315724.2014.907756 25738401

[255] Chang Y Zhai L Peng J Wu H Bian Z Xiao H. Phytochemicals as regulators of Th17/Treg balance in inflammatory bowel diseases. *Biomed Pharmacother.* (2021) 141:111931. 10.1016/j.biopha.2021.111931 34328111

[256] Löb S Königsrainer A Rammensee HG Opelz G Terness P. Inhibitors of indoleamine-2,3-dioxygenase for cancer therapy: can we see the wood for the trees?. *Nat Rev Cancer.* (2009) 9:445–52 10.1038/nrc2639 19461669

[257] Focaccetti C Izzi V Benvenuto M Fazi S Ciuffa S Giganti MG Polyphenols as immunomodulatory compounds in the tumor microenvironment: friends or foes? *Int J Mol Sci.* (2019) 20:1714. 10.3390/ijms20071714 30959898 PMC6479528

[258] Peyraud F Guegan JP Bodet D Cousin S Bessede A Italiano A. Targeting tryptophan catabolism in cancer immunotherapy era: challenges and perspectives. *Front Immunol.* (2022) 13:807271. 10.3389/fimmu.2022.807271 35173722 PMC8841724

[259] Noh KT Chae SH Chun SH Jung ID Kang HK Park YM Resveratrol suppresses tumor progression via the regulation of indoleamine 2,3-dioxygenase. *Biochem Biophys Res Commun.* (2013) 431:348–53 10.1016/j.bbrc.2012.12.093 23291179

[260] Jeong YI Kim SW Jung ID Lee JS Chang JH Lee CM. Curcumin suppresses the induction of indoleamine 2,3-dioxygenase by blocking the Janus-activated kinase-protein kinase Cdelta-STAT1 signaling pathway in interferon-gamma-stimulated murine dendritic cells. *J Biol Chem.* (2009) 284:3700–8. 10.1074/jbc.M807328200 19075017

[261] Jung ID Jeong Y-I Lee C-M Noh KT Jeong SK Chun SH COX-2 and PGE2 signaling is essential for the regulation of IDO expression by curcumin in murine bone row-derived dendritic cells. *Int Immunopharmacol.* (2010) 10:760–8. 10.1016/j.intimp.2010.04.006 20399909

[262] Ogawa K Hara T Shimizu M Nagano J Ohno T Hoshi M Ito H Tsurumi H (–)-Epigallocatechin gallate inhibits the expression of indoleamine 2,3-dioxygenase in human colorectal cancer cells. *Oncol Lett.* (2012) 4:546–50 10.3892/ol.2012.761 23741252 PMC3673646

[263] Chen S Corteling R Stevanato L Sinden J. Natural inhibitors of indoleamine 3,5-dioxygenase induced by interferon-gamma in human neural stem cells. *Biochem Biophys Res Commun.* (2012) 429:117–23. 10.1016/j.bbrc.2012.10.009 23063682

[264] Jeong YI Jung ID Lee JS Lee CM Lee JD Park YM. (–)-Epigallocatechin gallate suppresses indoleamine 2,3-dioxygenase expression in murine dendritic cells: evidences for the COX-2 and STAT1 as potential targets. *Biochem Biophys Res Commun.* (2007) 354:1004–9. 10.1016/j.bbrc.2007.01.076 17270146

[265] Hara T Matsumoto T Shibata Y Nakamura N Nakamura H Ninomiya S Prognostic value of the combination of serum l-kynurenine level and indoleamine 2,3-dioxygenase mRNA expression in acute myeloid leukemia. *Leuk Lymphoma.* (2016) 57:2208–11. 10.3109/10428194.2015.1128541 26762931

[266] El Kholy NM Sallam MM Ahmed MB Sallam RM Asfour IA Hammouda JA Expression of indoleamine 2,3-dioxygenase in acute myeloid leukemia and the effect of its inhibition on cultured leukemia blast cells. *Med Oncol.* (2011) 28:270–8. 10.1007/s12032-010-9459-6 20300979

[267] Nakamura N Hara T Shibata Y Matsumoto T Mabuchi R Nakamura H Combination of indoleamine 2,3-dioxygenase inhibitor and cytotoxic agents is a novel therapeutic option for non-hodgkin lymphoma. *Blood.* (2013) 122:4408

[268] Long GV Dummer R Hamid O Gajewski TF Caglevic C Dalle S Epacadostat plus pembrolizumab versus placebo plus pembrolizumab in patients with unresectable or metastatic melanoma (ECHO-301/KEYNOTE-252): a phase 3, randomised, double-blind study. *Lancet Oncol.* (2019) 20:1083–97 10.1016/S1470-2045(19)30274-8 31221619

[269] Sondak VK Khushalani NI. Echoes of a failure: what lessons can we learn? *Lancet Oncol.* (2019) 20:1037–9. 10.1016/S1470-2045(19)30312-2 31221617

[270] Emadi A Duong VH Pantin J Imran M Koka R Singh Z Indoximod combined with standard induction chemotherapy is well tolerated and induces a high rate of complete remission with MRD-negativity in patients with newly diagnosed AML: results from a phase 1 trial. *Blood.* (2018) 132(Suppl. 1):332. 10.1182/blood-2018-99-117433

[271] Zakharia Y McWilliams RR Rixe O Drabick J Shaheen MF Grossmann KF Phase II trial of the IDO pathway inhibitor indoximod plus pembrolizumab for the treatment of patients with advanced melanoma. *J Immunother Cancer.* (2021) 9:e002057. 10.1136/jitc-2020-002057 34117113 PMC8202104

[272] Yu CP Song YL Zhu ZM Huang B Xiao YQ Luo DY. Targeting TDO in cancer immunotherapy. *Med Oncol.* (2017) 34:73. 10.1007/s12032-017-0933-2 28357780

[273] Platten M Nollen EAA Röhrig UF Fallarino F Opitz CA. Tryptophan metabolism as a common therapeutic target in cancer, neurodegeneration and beyond. *Nat Rev Drug Discov.* (2019) 18:379–401. 10.1038/s41573-019-0016-5 30760888

[274] Hennequart M Pilotte L Cane S Hoffmann D Stroobant V Plaen E Constitutive IDO1 expression in human tumors is driven by cyclooxygenase-2 and mediates intrinsic immune resistance. *Cancer Immunol Res.* (2017) 5:695–709. 10.1158/2326-6066.CIR-16-0400 28765120

[275] Van den Eynde BJ van Baren N Baurain J-F. Is there a clinical future for IDO1 inhibitors after the failure of epacadostat in melanoma? *Annu Rev Cancer Biol.* (2020) 4:241–56. 10.1146/annurev-cancerbio-030419-033635

[276] Owczarek K Lewandowska U. The impact of dietary polyphenols on COX-2 expression in colorectal cancer. *Nutr Cancer.* (2017) 69:1105–18 29068698 10.1080/01635581.2017.1367940

[277] Ribeiro D Poença C Varela C Janela J Tavares da Silva EJ Fernandes E New phenolic cinnamic acid derivatives as selective COX-2 inhibitors. design, synthesis, biological activity and structure-activity relationships. *Bioorg Chem.* (2019) 91:103179. 10.1016/j.bioorg.2019.103179 31404794

[278] Lu Y Liu XF Liu TR Fan RF Xu YC Zhang XZ Celecoxib exerts antitumor effects in HL-60 acute leukemia cells and inhibits autophagy by affecting lysosome function. *Biomed Pharmacother.* (2016) 84:1551–57. 10.1016/j.biopha.2016.11.026 27884749

[279] Riva B De Dominici M Gnemmi I Mariani SA Minassi A Minieri V Celecoxib inhibits proliferation and survival of chronic myelogeous leukemia (CML) cells via AMPK-dependent regulation of β-catenin and mTORC1/2. *Oncotarget.* (2016) 7:81555–70. 10.18632/oncotarget.13146 27835591 PMC5348412

[280] Calgarotto AK Longhini AL Pericole de Souza FV Duarte ASS Ferro KP Santos I Immunomodulatory effect of green tea treatment in combination with low-dose chemotherapy in elderly acute myeloid leukemia patients with myelodysplasia-related changes. *Integr Cancer Ther.* (2021) 20:15347354211002647. 10.1177/15347354211002647 33754891 PMC7995304

[281] Mangaonkar A Mondal AK Fulzule S Pundkar C Park EJ Jillella A A novel immunohistochemical score to predict early mortality in acute myeloid leukemia patients based on indoleamine 2,3 dioxygenase expression. *Sci Rep.* (2017) 7:12892 10.1038/s41598-017-12940-0 29038460 PMC5643528

[282] Leisch M Greil R Pleyer L. IDO in MDS/AML disease progression and its role in resistance to azacitidine: a potential new drug target? *Br J Haematol.* (2020) 190:314–7. 10.1111/bjh.16710 32419137 PMC7496607

[283] Einsele H Briones J Ciceri F García-Cadenas I Falkenburg F Bolaños N Immune-based therapies for hematological malignancies: an update by the EHA SWG on immunotherapy of hematological malignancies. *HemaSphere.* (2020) 4:e423. 10.1097/HS9.0000000000000423 32904089 PMC7448369

[284] Sochacka-Ćwikła A Mączyński M Regiec AFDA-. approved drugs for hematological malignancies-the last decade review. *Cancers.* (2021) 14:87. 10.3390/cancers14010087 35008250 PMC8750348

[285] Wiernik PH. Alvocidib (flavopiridol) for the treatment of chronic lymphocytic leukemia. *Expert Opin Investig Drugs.* (2016) 25:729–34. 10.1517/13543784.2016.1169273 26998706

[286] Boffo S Damato A Alfano L Giordano A. CDK9 inhibitors in acute myeloid leukemia. *J Exp Clin Cancer Res.* (2018) 37:36. 10.1186/s13046-018-0704-8 29471852 PMC5824552

[287] Zeidner JF Foster MC Blackford AL Litzow MR Morris LE Strickland SA Randomized multicenter phase II study of flavopiridol (alvocidib), cytarabine, and mitoxantrone (FLAM) versus cytarabine/daunorubicin (7+3) in newly diagnosed acute myeloid leukemia. *Haematologica.* (2015) 100:1172–9. 10.3324/haematol.2015.125849 26022709 PMC4800702

[288] Litzow MR Wang XV Carroll MP Karp JE Ketterling RP Zhang Y A randomized trial of three novel regimens for recurrent acute myeloid leukemia demonstrates the continuing challenge of treating this difficult disease. *Am J Hematol.* (2019) 94:111–7. 10.1002/ajh.25333 30370956 PMC6298814

[289] Chen KTJ Militao GGC Anantha M Witzigmann D Leung AWY Bally MB. Development and characterization of a novel flavopiridol formulation for treatment of acute myeloid leukemia. *J Control Release.* (2021) 333:246–57. 10.1016/j.jconrel.2021.03.042 33798663

[290] Mirzaei H Bagheri H Ghasemi F Khoi JM Pourhanifeh MH Heyden YV Anti-cancer activity of curcumin on multiple myeloma. *Anticancer Agents Med Chem.* (2021) 21:575–86 32951583 10.2174/1871520620666200918113625

[291] Takada M Yamagishi K Iso H Tamakoshi A. Green tea consumption and risk of hematologic neoplasms: the Japan collaborative cohort study for evaluation of cancer risk (JACC Study). *Cancer Causes Control.* (2019) 30:1223–30. 10.1007/s10552-019-01220-z 31452000

[292] Hunstein W. Epigallocathechin-3-gallate in AL amyloidosis: a new therapeutic option? *Blood.* (2007) 110:2216. 10.1182/blood-2007-05-089243 17785589

[293] Mereles D Buss SJ Hardt SE Hunstein W Katus HA. Effects of the main green tea polyphenol epigallocatechin-3-gallate on cardiac involvement in patients with AL amyloidosis. *Clin Res Cardiol.* (2010) 99:483–90 10.1007/s00392-010-0142-x 20221615

[294] Palladini G Merlini G. What is new in diagnosis and management of light chain amyloidosis? *Blood.* (2016) 128:159–68 27053535 10.1182/blood-2016-01-629790

[295] Shanafelt TD Call TG Zent CS LaPlant B Bowen DA Roos M Phase I trial of daily oral Polyphenon E in patients with asymptomatic Rai stage 0 to II chronic lymphocytic leukemia. *J Clin Oncol.* (2009) 27:3808–14. 10.1200/JCO.2008.21.1284 19470922 PMC2727287

[296] Shanafelt TD Call T Zent CS LaPlant B Leis JF Bowen D Phase II trial of daily, oral green tea extract in patients with asymptomatic, Rai stage 0-II chronic lymphocytic leukemia (CLL). *J Clin Oncol.* (2010) 28:6522. 10.1200/jco.2010.28.15_suppl.6522

[297] Shanafelt TD Call TG Zent CS Leis JF LaPlant B Bowen DA Phase 2 trial of daily, oral Polyphenon E in patients with asymptomatic, Rai stage 0 to II chronic lymphocytic leukemia. *Cancer.* (2013) 119:363–70. 10.1002/cncr.27719 22760587 PMC3902473

[298] Willard PJ Damsker AJ Pickens PV. Effects of green tea on various types of indolent low grade b-cell lymphomas. *Blood.* (2020) 136:15–6. 10.1182/blood-2020-136346

[299] Orgel E Framson C Buxton R Kim J Li G Tucci J Caloric and nutrient restriction to augment chemotherapy efficacy for acute lymphoblastic leukemia: the IDEAL trial. *Blood Adv.* (2021) 5:1853–61 10.1182/bloodadvances.2020004018 33792627 PMC8045487

[300] Baron BW Thirman MJ Giurcanu MC Baron JM. quercetin therapy for selected patients with PIM1 Kinase-positive chronic lymphocytic leukemia/small lymphocytic lymphoma: a pilot study. *Acta Haematol.* (2018) 139:132–9. 10.1159/000486361 29444501

[301] Uckun FM Cogle CR Lin TL Qazi S Trieu VN Schiller G A phase 1B clinical study of combretastatin A1 diphosphate (OXi4503) and cytarabine (ARA-C) in combination (OXA) for patients with relapsed or refractory acute myeloid leukemia. *Cancers.* (2019) 12:74. 10.3390/cancers12010074 31888052 PMC7016810

[302] Shen H Shen J Pan H Xu L Sheng H Liu B Curcumin analog B14 has high bioavailability and enhances the effect of anti-breast cancer cells *in vitro* and *in vivo*. *Cancer Sci.* (2021) 112:815–27. 10.1111/cas.14770 33316116 PMC7894010

[303] He Y Li W Hu G Sun H Kong Q. Bioactivities of EF24, a novel curcumin analog: a review. *Front Oncol.* (2018) 8:614. 10.3389/fonc.2018.00614 30619754 PMC6297553

[304] Skoupa N Dolezel P Ruzickova E Mlejnek P. apoptosis induced by the curcumin analogue ef-24 is neither mediated by oxidative stress-related mechanisms nor affected by expression of main drug transporters ABCB1 and ABCG2 in human leukemia cells. *Int J Mol Sci.* (2017) 18:2289. 10.3390/ijms18112289 29088066 PMC5713259

[305] Olivera A Moore TW Hu F Brown AP Sun A Liotta DC Inhibition of the NF-κB signaling pathway by the curcumin analog, 3,5-Bis(2-pyridinylmethylidene)-4-piperidone (EF31): anti-inflammatory and anti-cancer properties. *Int Immunopharmacol.* (2012) 12:368–77 10.1016/j.intimp.2011.12.009 22197802 PMC3372981

[306] Kudo C Yamakoshi H Sato A Ohori H Ishioka C Iwabuchi Y Novel curcumin analogs, GO-Y030 and GO-Y078, are multi-targeted agents with enhanced abilities for multiple myeloma. *Anticancer Res.* (2011) 31:3719–26. 22110192

[307] Colomer R Sarrats A Lupu R Puig T. Natural polyphenols and their synthetic analogs as emerging anticancer agents. *Curr Drug Targets.* (2017) 18:147–59. 10.2174/1389450117666160112113930 26758667

[308] Karthikeyan A Senthil N Min T. Nanocurcumin: a promising candidate for therapeutic applications. *Front Pharmacol.* (2020) 11:487. 10.3389/fphar.2020.00487 32425772 PMC7206872

[309] Ventola CL. Progress in nanomedicine: approved and investigational nanodrugs. *P T.* (2017) 42:742–55. 29234213 PMC5720487

[310] Subramani PA Panati K Narala VR. Curcumin nanotechnologies and its anticancer activity. *Nutr Cancer.* (2017) 69:381–93. 10.1080/01635581.2017.1285405 28287321

[311] Mohan A Narayanan S Sethuraman S Krishnan UM. Novel resveratrol and 5-fluorouracil coencapsulated in PEGylated nanoliposomes improve chemotherapeutic efficacy of combination against head and neck squamous cell carcinoma. *Biomed Res Int.* (2014) 2014:424239 10.1155/2014/424239 25114900 PMC4119704

[312] Meng J Guo F Xu H Liang W Wang C Yang XD Combination therapy using co-encapsulated resveratrol and paclitaxel in liposomes for drug resistance reversal in breast cancer cells *in vivo*. *Sci Rep.* (2016) 6:22390 10.1038/srep22390 26947928 PMC4780086

[313] Sharifi-Rad J Quispe C Mukazhanova Z Knut E Turgumbayeva A Kipchakbayeva A Resveratrol-based nanoformulations as an emerging therapeutic strategy for cancer. *Front Mol Biosci.* (2021) 8:649395. 10.3389/fmolb.2021.649395 34540888 PMC8440914

[314] Chuan D Mu M Hou H Zhao N Li J Tong A Folic acid-functionalized tea polyphenol as a tumor-targeting nano-drug delivery system. *Mater Design.* (2021) 206:109805. 10.1016/j.matdes.2021.109805

[315] Hafeez U Parakh S Gan HK Scott AM. Antibody-drug conjugates for cancer therapy. *Molecules.* (2020) 25:4764. 10.3390/molecules25204764 33081383 PMC7587605

[316] Pennesi E Michels N Brivio E van der Velden VHJ Jiang Y Thano A Inotuzumab ozogamicin as single agent in pediatric patients with relapsed and refractory acute lymphoblastic leukemia: results from a phase II trial. *Leukemia.* (2022) 36:1516–24. 10.1038/s41375-022-01576-3 35468945 PMC9162924

[317] Nirachonkul W Ogonoki S Thumvijit T Chiampanichayakul S Panyajai P Anuchapreeda S CD123-targeted nano-curcumin molecule enhances cytotoxic efficacy in leukemic stem cells. *Nanomaterials.* (2021) 11:2974 10.3390/nano11112974 34835741 PMC8620973

[318] Langone P Debata PR Dolai S Curcio GM Inigo JDR Raja K Coupling to a cancer cell-specific antibody potentiates tumoricidal properties of curcumin. *Int J Cancer.* (2012) 131:E569–78. 10.1002/ijc.26479 21989768

[319] Wagner H Efferth T. Introduction: novel hybrid combinations containing synthetic or antibiotic drugs with plant-derived phenolic or terpenoid compounds. *Phytomedicine.* (2017) 37:1–3. 10.1016/j.phymed.2017.10.020 29174652

[320] Dana MP Sadoughi F Asemi Z. Yousefi B The role of polyphenols in overcoming cancer drug resistance: a comprehensive review. *Cell Mol Biol Lett.* (2022) 27:1. 10.1186/s11658-021-00301-9 34979906 PMC8903685

[321] Domínguez-Martín EM Díaz-Lanza AM Faustino CMC. Anticancer hybrid combinations: mechanisms of action, implications and future perspectives. *Curr Pharm Des.* (2018) 24:4312–33. 10.2174/1381612825666190110162529 30636587

[322] Zhou Y Wang R Chen B Sun D Hu Y Xu P. Daunorubicin and gambogic acid coloaded cysteamine-CdTe quantum dots minimizing the multidrug resistance of lymphoma *in vitro* and *in vivo*. *Int J Nanomed.* (2016) 11:5429–42. 10.2147/IJN.S115037 27799767 PMC5077128

[323] Teiten MH Dicato M Diederich M. Hybrid curcumin compounds: a new strategy for cancer treatment. *Molecules.* (2014) 19:20839–63. 10.3390/molecules191220839 25514225 PMC6271749

[324] Mahbub AA Maitre CLL Haywood-Small S Cross NA Jordan-Mahy N. Polyphenols enhance the activity of alkylating agents in leukaemia cell lines. *Oncotarget.* (2019) 10:4570–86 31360305 10.18632/oncotarget.27068 PMC6642044

[325] Cramer H Cohen L Dobos G Witt CM. Integrative oncology: best of both worlds-theoretical, practical, and research issues. *Evid Based Complement Alternat Med.* (2013) 2013:383142. 10.1155/2013/383142 24371456 PMC3863498

[326] Hatami E Jaggi M Chauhan SC Yallapu MM. Gambogic acid: a shining natural compound to nanomedicine for cancer therapeutics. *Biochim Biophys Acta Rev Cancer.* (2020) 1874:188381. 10.1016/j.bbcan.2020.188381 32492470 PMC7484097

[327] Liu N Huang H Xu L Hua X Li X Liu S The combination of proteasome inhibitors bortezomib and gambogic acid triggers synergistic cytotoxicity *in vitro* but not *in vivo*. *Toxicol Lett.* (2014) 224:333–40. 10.1016/j.toxlet.2013.11.021 24291039

[328] Zhang W Qiao L Wang X Senthilku R Wang F Chen B. Inducing cell cycle arrest and apoptosis by dimercaptosuccinic acid modified Fe3O4 magnetic nanoparticles combined with nontoxic concentration of bortezomib and gambogic acid in RPMI-8226 cells. *Int J Nanomed.* (2015) 10:3275–89. 10.2147/IJN.S80795 25995634 PMC4425315

[329] Chi Y Zhan XK Yu H Xie GR Wang ZZ Xiao W An open-labeled, randomized, multicenter phase IIa study of gambogic acid injection for advanced malignant tumors. *Chin Med J.* (2013) 126:1642–6. 23652044

[330] Banik K Harsha C Bordoloi D Lalduhsaki Sailo B Sethi G Leong HC Therapeutic potential of gambogic acid, a caged xanthone, to target cancer. *Cancer Lett.* (2018) 416:75–86. 10.1016/j.canlet.2017.12.014 29246645

[331] Liu Y Chen Y Lin L Li H. Gambogic acid as a candidate for cancer therapy: a review. *Int J Nanomedicine.* (2020) 15:10385–99. 10.2147/IJN.S277645 33376327 PMC7764553

[332] Li M Su F Zhu M Zhang H Wei Y Zhao Y Research progress in the field of gambogic acid and its derivatives as antineoplastic drugs. *Molecules.* (2022) 27:2937. 10.3390/molecules27092937 35566290 PMC9102264

[333] Wang F Dong L Wei X Wang Y Chang L Wu H Effect of gambogic acid-loaded porous-lipid/PLGA microbubbles in combination with ultrasound-triggered microbubble destruction on human glioma. *Front Bioeng Biotechnol.* (2021) 9:711787. 10.3389/fbioe.2021.711787 34604184 PMC8479098

[334] de Carvalho JTG Da Silva Baldivia D de Castro DTH Dos Santos HF Dos Santos CM Oliveira AS The immunoregulatory function of polyphenols: implications in cancer immunity. *J Nutr Biochem.* (2020) 85:108428. 10.1016/j.jnutbio.2020.108428 32679443

[335] Mileo AM Nisticò P Miccadei S. Polyphenols: immunomodulatory and Therapeutic Implication in Colorectal Cancer. *Front Immunol.* (2019) 10:729. 10.3389/fimmu.2019.00729 31031748 PMC6470258

[336] Xu L Zhang Y Tian K Chen X Zhang R Mu X Apigenin suppresses PD-L1 expression in melanoma and host dendritic cells to elicit synergistic therapeutic effects. *J Exp Clin Cancer Res.* (2018) 37:261. 10.1186/s13046-018-0929-6 30373602 PMC6206930

[337] Jiang ZB Wang WJ Xu C Xie YJ Wang XR Zhang YZ Luteolin and its derivative apigenin suppress the inducible PD-L1 expression to improve anti-tumor immunity in KRAS-mutant lung cancer. *Cancer Lett.* (2021) 515:36–48. 10.1016/j.canlet.2021.05.019 34052328

[338] Shao Y Zhu W Da J Xu M Wang Y Zhou J Bisdemethoxycurcumin in combination with α-PD-L1 antibody boosts immune response against bladder cancer. *Onco Targets Ther.* (2017) 10:2675–83. 10.2147/OTT.S130653 28579805 PMC5449128

[339] Mohan A Narayanan S Sethuraman S Krishnan UM. Combinations of plant polyphenols & anti-cancer molecules: a novel treatment strategy for cancer chemotherapy. *Anticancer Agents Med Chem.* (2013) 13:281–95 22721388 10.2174/1871520611313020015

[340] O’Brien S Kay NE. Maintenance therapy for B-chronic lymphocytic leukemia. *Clin Adv Hematol Oncol.* (2011) 9:22–31 21326143

